# Photochemically-driven highly efficient intracellular delivery and light/hypoxia programmable triggered cancer photo-chemotherapy

**DOI:** 10.1186/s12951-023-01774-w

**Published:** 2023-01-12

**Authors:** Wei Zhang, Cuncheng Zhang, Chao Yang, Xingyue Wang, Weiwei Liu, Mi Yang, Yang Cao, Haitao Ran

**Affiliations:** 1 Chongqing Key Laboratory of Ultrasound Molecular Imaging & Department of Ultrasound, Second Affiliated Hospital of Chongqing Medical University, No. 74 Linjiang Rd, Yuzhong District, Chongqing, 400010 People’s Republic of China; 2 Department of Ultrasound, Chongqing General Hospital, No. 104, Pipashan Main Street, Yuzhong District, Chongqing, 40013 China; 3 Department of Radiology, Chongqing General Hospital, No. 104, Pipashan Main Street, Yuzhong District, Chongqing, 40013 China

**Keywords:** Porphyrinic metal organic framework, Stimuli-responsive, Hypoxia, Nanomedicine, Photochemotherapy

## Abstract

**Background:**

Using nanotechnology to improve the efficiency of tumor treatment represents a major research interest in recent years. However, there are paradoxes and obstacles in using a single nanoparticle to fulfill all the requirements of complex tumor treatment.

**Results:**

In this paper, a programmed-triggered nanoplatform (APP NPs), which is sequentially responsive to light and hypoxia, is rationally integrated for photoacoustic (PA) imaging-guided synergistic cancer photo-chemotherapy. The nanoplatform is constructed by in situ hybridization of dopamine monomer in the skeleton of PCN-224 and loading prodrug banoxantrone (AQ4N). Upon first-stage irradiation with a 660 nm laser, cellular internalization was effectively promoted by a photosensitizer-mediated photochemical effect. Furthermore, under second-stage irradiation, APP NPs exhibit a notably high photothermal conversion efficiency and sufficient reactive oxygen species (ROS) production for photothermal therapy (PTT) and photodynamic therapy (PDT), respectively, which not only triggers rapid intercellular drug release but also consequently aggravates tumor hypoxia levels, and aggravated hypoxia can further active the cytotoxicity of AQ4N for chemotherapy. Both in vitro and in vivo studies confirm that the dual-stage light guided photo-chemotherapy strategy exhibits a greatly enhanced anticancer effects and superior therapeutic safety.

**Conclusion:**

This work represents a versatile strategy to construct a dual-stage light induced PDT/PTT and hypoxia-activated chemotherapy nanoplatform and will be promising for the development of multistimuli-responsive nanosystems with programmable functions for precise cancer therapy.

**Supplementary Information:**

The online version contains supplementary material available at 10.1186/s12951-023-01774-w.

## Introduction

Autologous dysregulation, as well as neoplasm and irregular vasculature, results in abnormal physicochemical tumor microenvironment (TME) signal release (featuring hypoxia, increased levels of some biomarkers, acidic pH, enhanced reactive oxygen species); drives drug resistance, invasion and metastasis; compromises the outcome of cancer therapy; and is responsible for the majority of clinical recrudesces and deaths [1–3]. To utilize the pathological cues for precise treatment, intelligent nanomaterials capable of degradation or conformation changes have been devised with specific responses to external stimuli (light, ultrasound, temperature) or endogenous stimuli or signals (hypoxia, pH, H_2_O_2_) for targeted delivery and on-demand drug release in tumor sites, which could significantly enhance anticancer efficacy and reduce off-target and adverse side effects [4, 5]. However, despite the improved therapeutic efficacy, most combinatorial therapies require a combination of drugs, antibodies, inhibitors, and gene strands to be used in concert and released in sequence at the right time and place, which is a formidable challenge with these single-stimulus responsive nanoplatforms [6, 7]. Thus, multistimuli-responsive nanosystems with programmable functions to combat multiple biological barriers are urgently needed to enhance spatiotemporal controllability for activated targets and overcome the intrinsic drawbacks of single-stimulus responsive systems.

Photo-based therapeutic approaches such as PDT and PTT have garnered extensive attention as cancer treatment owing to their noninvasiveness, controllability, localized treatment, high spatiotemporal precision, and negligible drug resistance [8–11]. However, because PDT strictly depends on the availability of tissue oxygen to produce ^1^O_2_, continuously utilizing tissue oxygen and the PDT-mediated effects of shutting down vasculature would further exacerbate hypoxia and in turn limit PDT efficiency [12–16]. PTT is a without relying on oxygen treatment paradigm that destroy tumor cells via local hyperthermia triggered by near infrared (NIR) light-activated photothermal agent [17–19]. Although PTT is effective method for tumor ablation, it has the disadvantage of possibly causing radiation damage to the skin; in addition, tumor regrowth/reoccurrence, which is caused by inadequate hyperthermia and uneven heat delivery within the tumor region, may occur [20, 21]. It is suggested that combined PDT and PTT can boost the ^1^O_2_ production required for PDT [22]. Importantly, it was found that PTT could improve the tumor reoxygenation level of TME by dilating blood vessels and increasing intra-tumoral blood flow [23]. In addition, photochemical internalization (PCI) caused by PDT can increase the permeability of the cell membrane and increased uptake of nanoparticles by the cell, and intracellular delivery may be activated by external light sources (for example, 600–800 nm light) that optimally penetrate the target tissue and can cover solid tumors more efficiently [24]. In view of these outcomes, rather than attempting to overcome hypoxia, the strategy of introducing hypoxia-activatable prodrugs merits serious consideration. For instance, AQ4N is a novel bio-reductive prodrug that can be activated to toxic AQ4 for bio-reductive chemotherapy under hypoxia [25, 26]. Therefore, in combination with oxygen-consuming phototherapy and photo-aggravated hypoxia-activated prodrug may be a more effective tactics to strengthen the traditional phototherapy and minimize the potential phototoxicity in preclinical models.

In recent years, metal-organic frameworks (MOFs), composed of metal ions/clusters linked by organic bridging ligands, have attracted tremendous attention in many applications, including catalysis, gas storage, sensing, and drug delivery [27–30], as their metal ions and organic linkers can be highly tailorable. Owing to their high drug loading ability, intrinsic biodegradability, structural/compositional tunability and controlled size/shapes [31, 32], MOFs present tremendous potential as precursors for the preparation of nanomaterials for biomedical applications. However, the poor biocompatibility and potential toxicity of MOFs limit their in vivo applications as drug carriers for tumor therapy. Polydopamine (PDA), prepared by the self-oxidation of the catecholamine neurotransmitter dopamine, has been widely investigated due to its sample synthesis, high photothermal conversion efficiency, and good biocompatibility [33, 34]. Given its wide absorption profile and nonradiative transition, PDA has been investigated as a photoacoustic contrast agent [35]. More importantly, the polymerization procedure of dopamine forms PDA coatings around the surfaces of many organic, inorganic and biological materials of various dimensions, providing a convenient means for surface modification [36, 37].

**Fig. 1:**
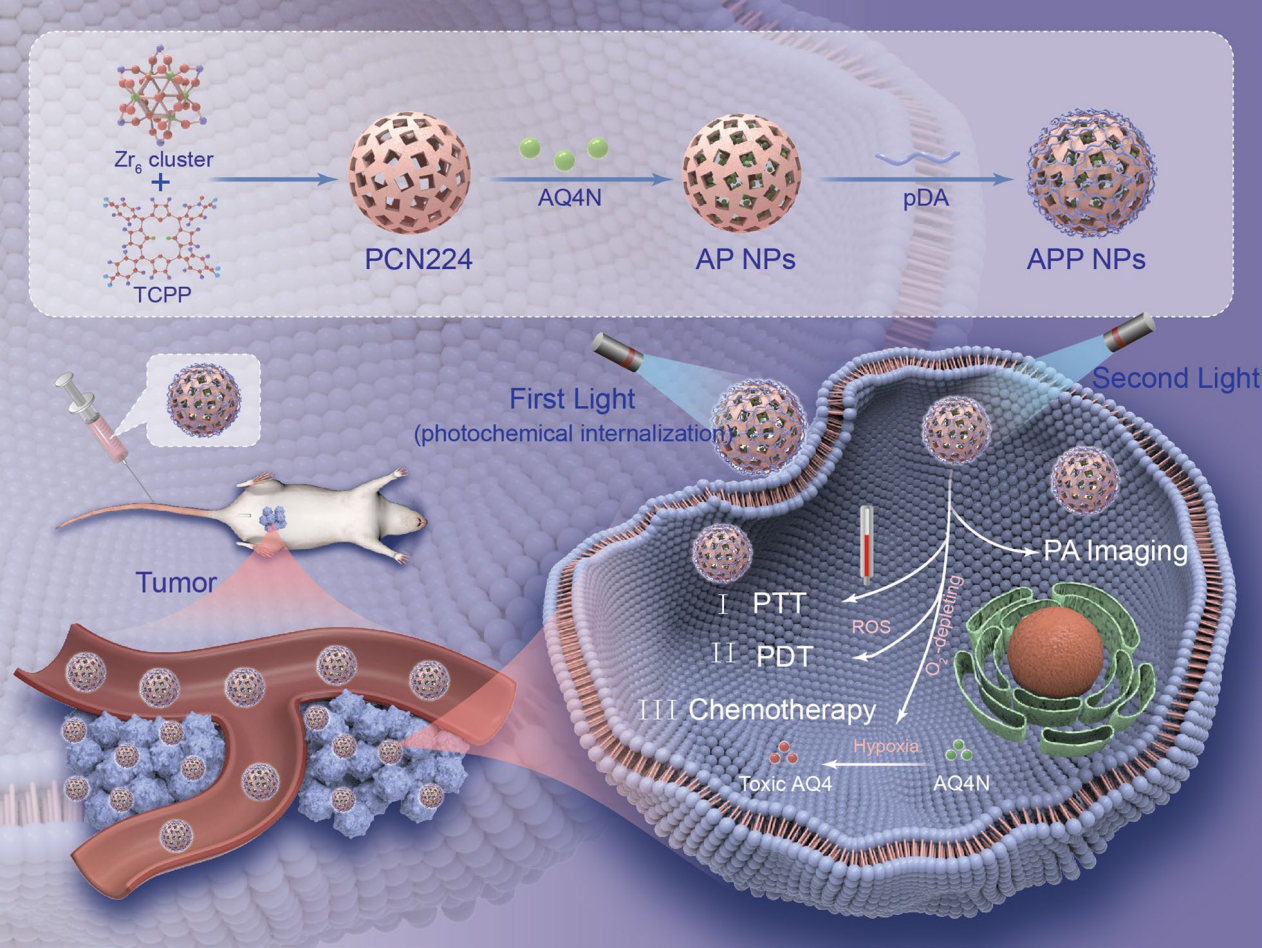
Schematic illustration of APP NPs for dual-stage light-guided highly efficient intracellular delivery and light/hypoxia programmable triggered cancer photo-chemotherapy

In this work, we rationally integrated a porphyrin-based MOF and hypoxia-sensitive AQ4N prodrug to form a programmed-triggered nanosystem for enhanced tumor photochemotherapy (Fig. 1). Herein, we selected PCN-224, which has a high photosensitizer (PS) loading ability, can induce the facile diffusion of singlet oxygen and can avoid the self-quenching of porphyrin fluorescence, as a template. Hypoxia activated prodrug AQ4N were efficiently loaded into the metal organic framework PCN-224 to yield AQ4N@PCN-224. Then, PDA was coated on the surface of MOFs to form AQ4N@PCN-224@PDA (APP NPs) that have improved stability and dispersibility in the systemic circulation. Overall, the multifunctional nanosystem exhibits five important properties, which also form the rationale behind the design: (i) facile self-assembly of simple nontoxic components that interact together to produce a multistimuli-responsive and highly biocompatible nanoplatform; (ii) utilizing dual-stage irradiation with a 660 nm laser, on the one hand, promotes the cellular uptake of nanoparticles by PCI, and on the other hand, it provides a safe therapeutic approach with enhanced curative effect within a short treatment time; (iii) combined with PDT and PTT, the local hyperthermia produced by PDA, could observably improve the blood flow speed of tumor tissues and further improve the oxygen level to enhance PDT efficacy, which was crucial to reduce laser power and irradiation time; (iv) with the photoinduced elevated hypoxia, the released AQ4N is reduced into the cytotoxic AQ4 form to accomplish an amplified chemotherapeutic effect; (v) under the guidance of precise and noninvasive PA imaging, the safety and effectiveness of the therapeutic process were at a high level. Therefore, the nanosystem have considerable potential as a tumor theranostic platform to realize imaging-guided and multistimuli-responsive cancer photochemotherapy.

## Results and discussion

### Design, synthesis, and characterization of APP NPs

**Fig. 2:**
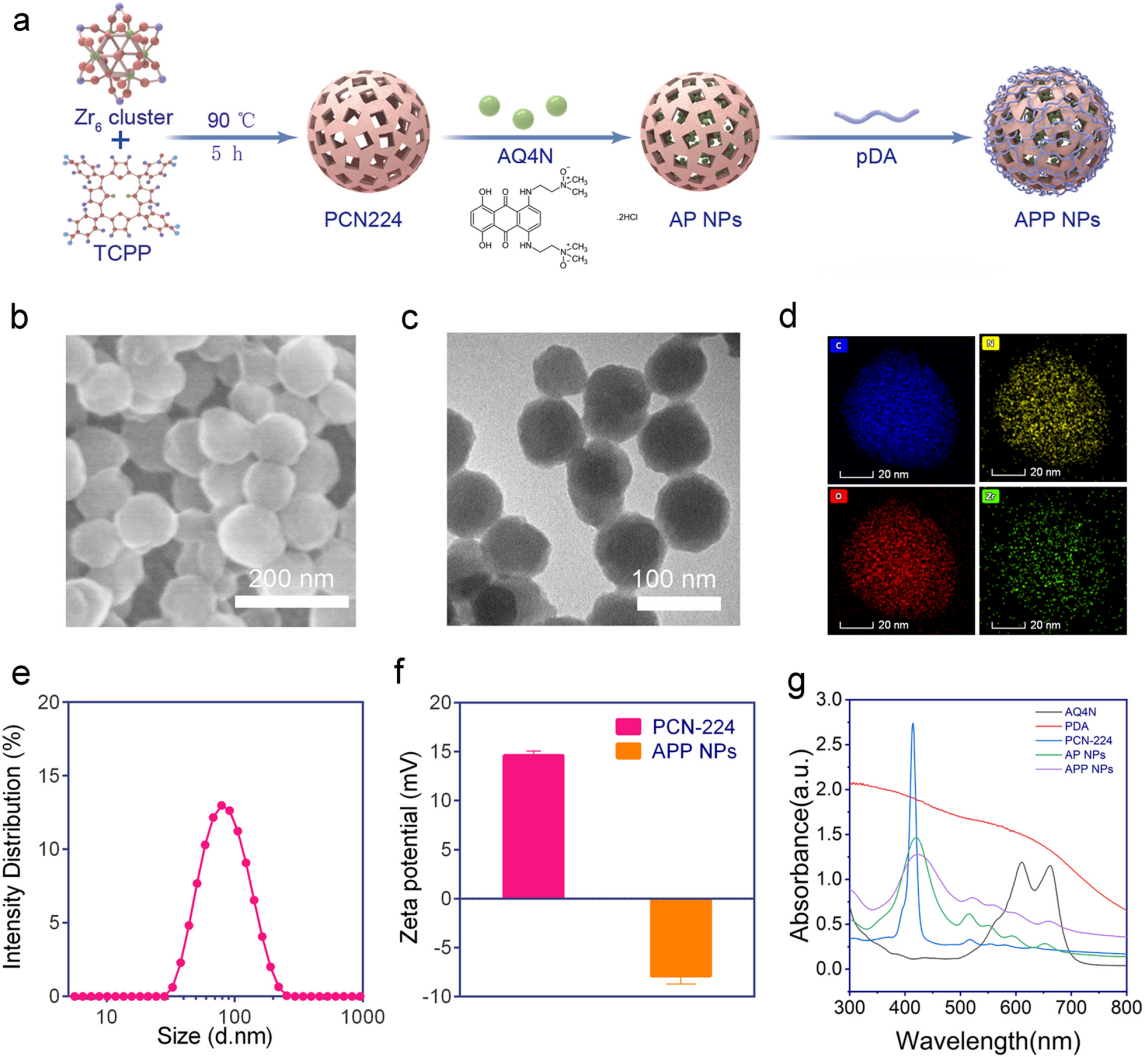
Preparation and characterization of APP NPs. **a** Preparation of APP NPs. **b** SEM and (**c**) TEM of APP NPs. **d** Elemental distribution mapping of APP NPs. **e** Size distribution of APP NPs. **f** Zeta potential of PCN-224 and APP NPs. **g** UV–vis spectrum of AQ4N, PDA, PCN-224, AP NPs, and APP NPs

In this work, APP NPs were prepared in three steps, as clearly illustrated in Fig. 2a. First, we synthesized PCN-224 using a solvothermal method according to previous studies [38]. Subsequently, after loading AQ4N, nanoparticles were dispersed into freshly prepared dopamine solutions in Tris buffer at pH 8.5. As seen in Fig. 2b, c, the as-obtained APP NPs exhibited a uniform spherical morphology and good monodispersity with a size of 90 nm. When coated with PDA, the rough surface and improved dispersibility of APP NPs indicates that the dopamine monomer had been polymerized into the pores and surfaces of PCN-224 (Additional file1: Fig. S1). In addition, after reacting with a certain proportion of dopamine, the color of the suspension totally changed from purple to black (Additional file 1: Fig. S2), indicating the successful polymerization of the dopamine monomer. The energy-dispersive X-ray elemental mapping images (Fig. 2d; Additional file 1: Fig. S3) of the typical products showed an even distribution of C (blue), N (yellow), Zr (green) and O (red) throughout the whole nanoparticle. Dynamic light scattering measurements indicated that the APP NPs possessed an average hydrodynamic diameter of 105 nm (Fig. 2e), and the mean particle size did not show appreciable change within 7 days (Additional file 1: Fig. S4), suggesting that the APP NPs potential had a passive targeting ability through the enhanced permeation and retention effect and excellent long-term stability. Moreover, the zeta potentials of the as-synthesized PCN-224 and APP NPs were 14.6 ± 0.4 mV and − 7.8 ± 0.8 mV, respectively (Fig. 2f), and the change of potential was attributed to the electronegativity of PDA. Figure 2 g shows the typical UV–vis absorption of AQ4N, PDA, PCN-224, AP NPs, and APP NPs. The specific surface area and porosity of PCN-224 were evaluated by N_2_ absorption-desorption isotherms and pore-size distribution analysis (Additional file 1: Figs. S5–S7). The PCN-224 possesses a high Brunauer-Emmett-Teller specific surface area of 394.2 m^2^ g^−1^ and a uniform pore size and 4.5 nm, which were conductive to the efficient loading of AQ4N. The PCN-224 after modification shows much lowered surface area, which is due to the polymerization of PDA. The APP NPs provided direct evidence for the persistence of an absorption peak at 500–700 nm for PCN-224, and the peaks affiliated to methylene in PDA appeared in the FTIR spectrum of PP NPs (Additional file 1: Fig. S8). These results further confirming the successful integration of PDA to PCN-224. The encapsulation efficiency and loading efficiency of AQ4N are 62.35 ± 2.17% and 15.12 ± 3.51%, respectively.

### Photothermal and photodynamic performance of APP NPs

**Fig. 3:**
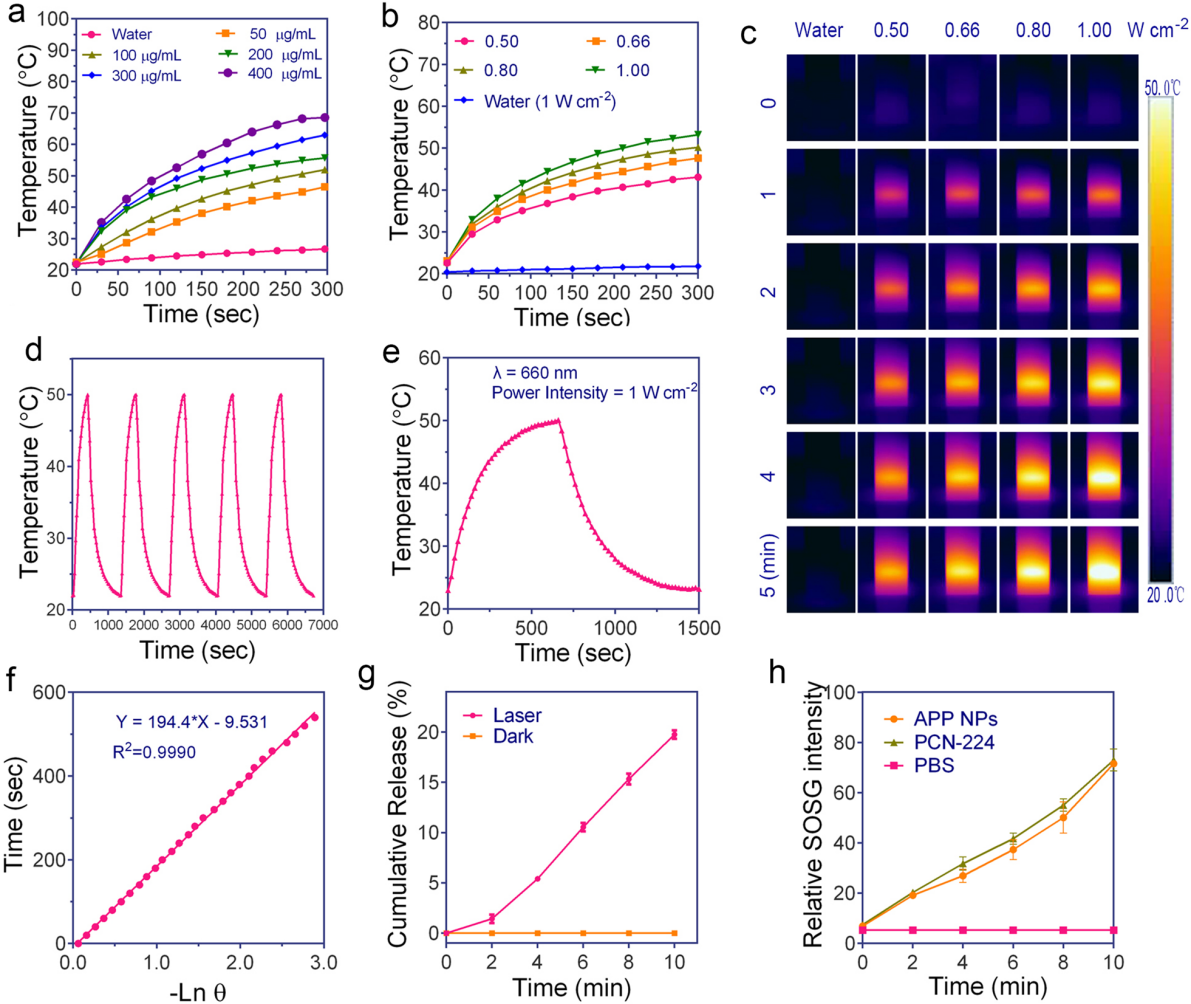
**a** Heat curves of APP NPs solution with different concentrations. **b**, **c** Heat curves and infrared thermographic images of APP NPs (100 µg mL^−1^) upon 660 nm laser exposure at various power densities. **d** Photothermal stability of APP NPs for five successive cycles of on/off laser irradiation. **e** Heat curves of APP NPs solution over a period of 1500 s versus the concentration of 50 µg mL^−1^. **f** The time constant was calculated form the cooling period. **g** Drug release profiles of APP NPs under different treatments. **h** The relative SOSG intensity of different nanoparticles after irradiation for different time durations

Herein, a single wavelength NIR irradiation laser was used as a photoenergy source, inducing the generation of heat and ROS by APP NPs. PDA modification not only reduced the toxicity but also endowed the APP NPs with a photothermal conversion ability [37, 39]. The APP NPs exhibited good photothermal properties. Compared with water, the temperature of APP NPs with various concentrations rapidly increased (Fig. 3a), revealing a concentration-dependent manner upon the same laser irradiation (0.8 W cm^−2^, 5 min). As shown in Fig. 3b and c, when the concentration of APP NPs was 100 µg mL^−1^, the temperature could increase from 22 to 53 °C within 300 s with a laser intensity of 1.0 W cm^−2^. In addition, there was no obvious change in temperature elevation of APP NPs, after 5 irradiation/cooling cycles (Fig. 3d), validating that they have perfect photostability under irradiation. The photothermal conversion efficiency of the APP NPs was calculated to be 30% (Fig. 3e, f), which was superior to those of existing photothermal agents such as gold nanorods and cyanine dyes (26.6% [40]). Such an enhanced photothermal effect by nanoparticles may originate from π-π stacking interactions between the polarizable π electron cloud of –C≡N– linkers and PDA, resulting in the enhancement of electron density in APP NPs [32].

Stimulated by hyperthermia upon 660 nm laser irradiation for 10 min, 19.75 ± 0.41% of the encapsulated AQ4N molecules were gradually released from the APP NPs (Fig. 3g), whereas no significant release of AQ4N was observed without irradiation, demonstrating photothermal-induced drug release.

The ^1^O_2_ generation capability of APP NPs under laser irradiation was evaluated by singlet oxygen sensor green (SOSG), a highly sensitive ^1^O_2_ indicator. The durability of singlet oxygen generated by APP NPs was monitored during a 10 min irradiation, as shown in Fig. 3h. With the extension of irradiation time, the APP NPs exhibited obviously higher ROS production efficacy, which is almost comparable to that of PCN-224, indicating the excellent ability of APP NPs to continuously produce singlet oxygen and further PDT potential against cancer. The electron spin resonance (ESR) spectrum was used to further investigate the ROS produced by APP NPs after irradiated with laser. As shown in Additional file 1: Fig. S9, a higher ESR signal was achieved at prolonged time duration after irradiated with 660 nm laser. Collectively, APP NPs are able to produce both conspicuous photothermal effects and singlet oxygen under light irradiation, validating that APP NPs are potential theranostic agents for PDT/PTT under a single wavelength laser.

### PCI-induced internalization measurement

**Fig. 4:**
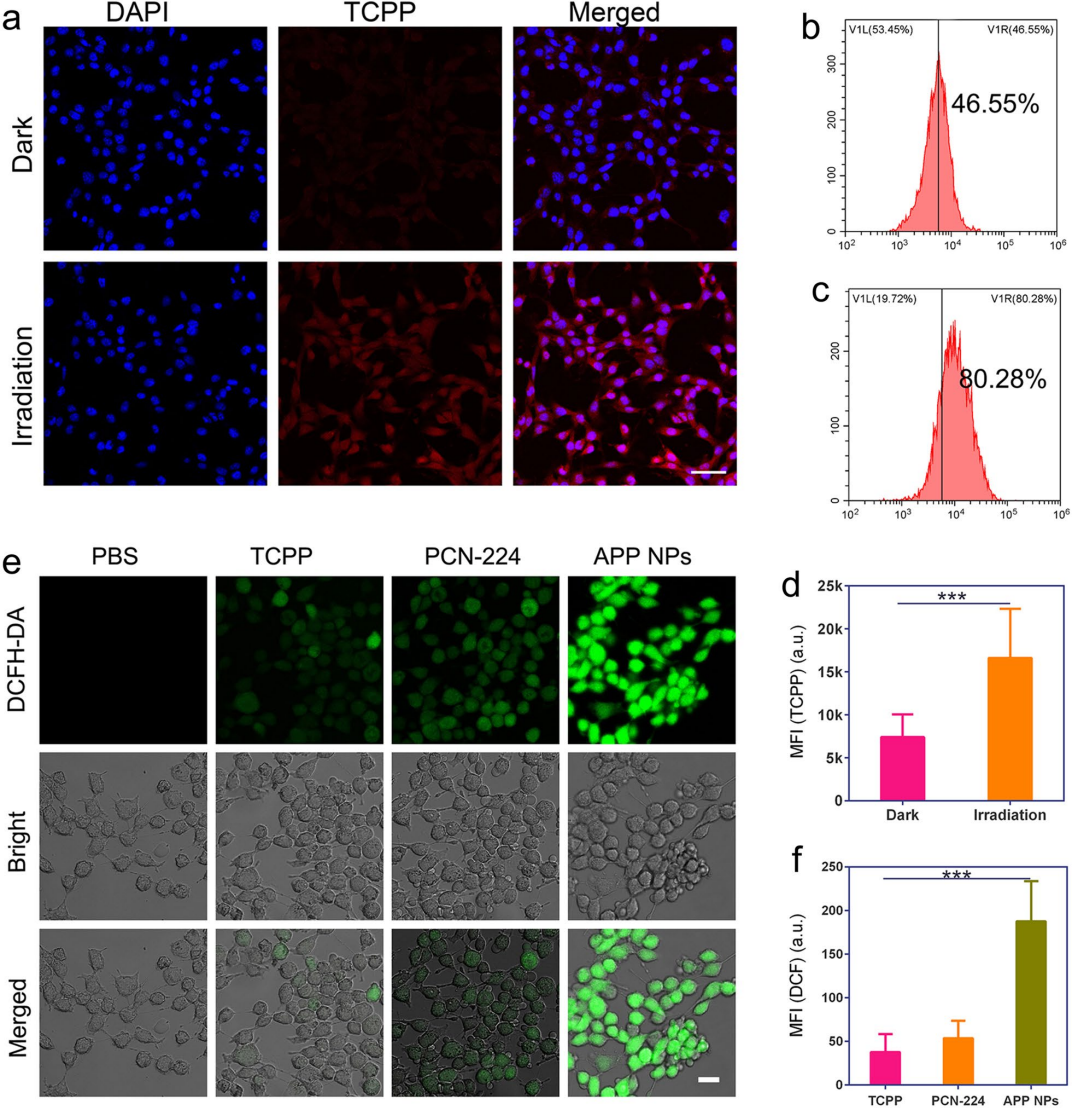
**a**–**c** CLSM images and flow cytometry analysis of the intracellular localization of APP NPs with or without light irradiation. (Scale bar: 50 μm) **d** MFI of TCPP for quantitative cellular uptake of APP NPs (n = 3). **e** CLSM of ROS production in 4T1 cells incubated with NPs upon light irradiation (scale bar: 10 μm). **f** MFI of DCF for the quantitative analysis of intracellular ROS levels (n = 3) *** *p* < 0.01

Improving the uptake of nanoparticles by tumor cells is crucial for cancer therapy. A relatively low dose of 660 nm NIR light (20 mW cm^−2^, 2 min) was used to evaluate the PCI effect on the cellular uptake of APP NPs. As shown in Fig. 4a, the red fluorescence signal of Tetrakis (4-carboxyphenyl) porphyrin (TCPP) could be clearly observed in the cytoplasm of 4T1 cells after exposure to a low dose of laser irradiation. The quantitative internalization efficiency of APP NPs was further investigated using flow cytometry. The results (Fig. 4b, c) verified that a low dose laser irradiation can enhance cellular uptake efficiency (from 46.55 to 80.28%). In addition, the mean fluorescence intensity (MFI) of TCPP in cells treated with near-infrared light was elevated by 2.5-fold compared with that in dark treatment (Fig. 4d). These results suggest that PCI induces effectively cellular internalization of APP NPs.

### Intracellular ROS generation by PDT

The ROS production ability of APP NPs in tumor cells was also measured using 2’,7’-dichlorofluorescein diacetate (DCFH-DA) as the indicator [41, 42]. DCFH-DA has no fluorescence itself but can be rapidly oxidized into fluorescent 2’7’-dichlorofluorescein (DCF) by ROS to emit green fluorescence in living cells. As shown in Fig. 4e, **4T1** cells in the APP NPs plus light group exhibited strong green fluorescence, indicating the mass generation of ROS from APP NPs in tumor cells after light irradiation. By comparison, weak fluorescence signals were displayed in the other groups: TCPP + Light and PCN-224 + Light. The TCPP + Light and PCN-224 + Light groups caused only a certain amount of ROS generation, which might be related to their poor solubility in the aqueous solution. The relative fluorescence intensity in the APP NPs plus light group was significantly higher than that in any other group (Fig. 4f), which was consistent with that detected by CLSM. In addition, the flow cytometry also substantiated the effective light-activated ROS production ability of APP NPs, as shown in Additional file 1: Fig. S10. Next, we evaluated the APP NPs caused hypoxia in vitro by western blot assay and fluorescent probe. As shown in Additional file 1: Figs. S11 and S12, cells incubated with APP NPs showed significant HIF-α protein expression and hypoxia signals after irradiated with 660 nm laser, which demonstrated that APP NPs could induce intracellular hypoxia upon laser irradiation.

### In vitro synergistic therapeutic efficacy of APP NPs

**Fig. 5:**
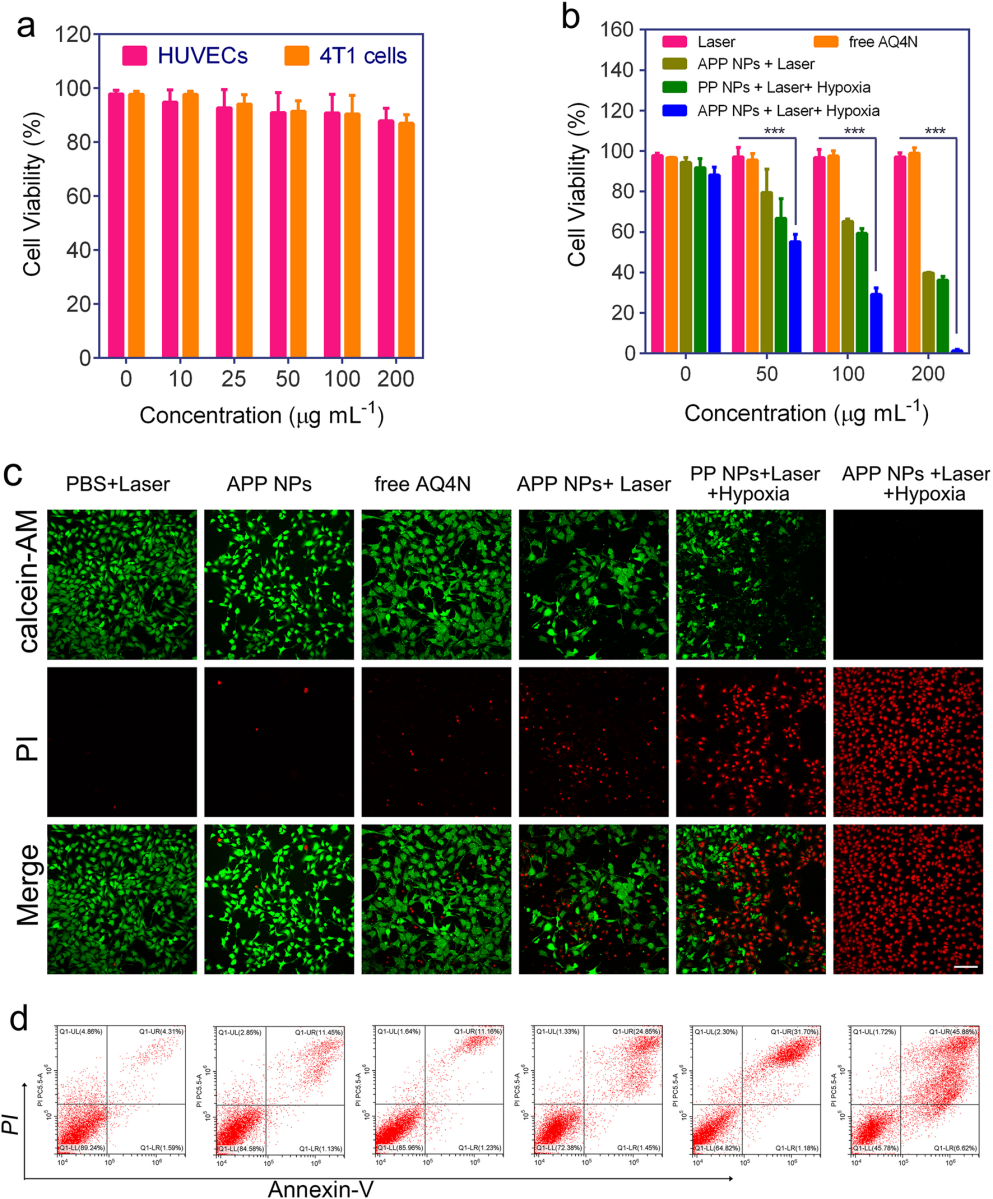
**a** Cell viability of HUVECs and 4T1 cells co-cultured with APP NPs for 24 h at different concentrations. **b** Cell viability of 4T1 cells co-cultured with NPs followed by various treatments. **c** Dead/live cell staining of 4T1 cells treated with PBS, free AQ4N, PP NPs and APP NPs followed by various administration. The scale bar is 50 μm. **d** Flow cytometry analysis of 4T1 cells co-cultured with PBS, free AQ4N, PP NPs and APP NPs followed by various treatments

First, the viability of Human umbilical vein endothelial cells (HUVECs) and 4T1 cells was tested after co-cultured with various concentrations of APP NPs for 24 h in dark. As shown in Fig. 5a, no obvious toxicity of APP NPs to HUVECs and 4T1 cells was detected even at concentration up to 200 µg mL^−1^, which suggested that the APP NPs had a very low dark cytotoxicity and fantastic biocompatibility. Since APP NPs could have PDT/PTT effects under single laser irradiation (660 nm) and hypoxia-induced chemotherapy ability after reducing the prodrug AQ4N to AQ4 by photoinduced hypoxia, the in vitro synergistic antitumor performance of APP NPs under aerobic and hypoxic conditions was quantitatively assessed. As a result, APP NPs exhibited considerably enhanced growth inhibition ability (88.3%) under laser irradiation and hypoxic conditions (Fig. 5b) but inhibited only 49% of cell proliferation without laser irradiation; moreover, PCN224@PDA (PP NPs)-treated cells inhibited only 43% of cell proliferation under laser irradiation and hypoxic conditions. Thus, since hypoxia further enhanced the cytotoxicity of AQ4N, the low cell viability of the APP NP treatment group upon laser irradiation and hypoxic conditions indicated the powerful synergistic anticancer effect of our APP NPs compared with the limited therapeutic effect against cancer cells in other groups. In addition, the single/dual-stage light synergetic therapeutic effect of APP NPs were assessed against 4T1 cells. As revealed in Additional file 1: Fig. S13, APP NPs with dual-stage light treatment demonstrated a significantly higher toxicity than that of APP NPs with single light irradiation, and the obviously enhanced treatment effect might be attributed to the first-stage light-driven highly efficient intracellular delivery of nanoparticles [43].

Annexin V-FITC/PI assay was performed to investigate the mechanism involved in the enhanced antitumor efficacy of APP NPs in 4T1 cells. As presented in Fig. 5d, the proportion of apoptosis cells in laser group/APP NPs group was less than 14%. suggesting the negligible phototoxicity of laser and nanoparticle itself. By comparison, the proportion of apoptosis cells significantly increased to 22% in the APP NPs + Laser group. More importantly, once the APP NPs-treated 4T1 cells were performed with laser and hypoxic, proportion of apoptosis cells significantly increased to 35%, which was significantly higher than that of the PP NPs-treated cells with irradiation (23%), resulting from hypoxia-triggered chemocytotoxicity of AQ4N. In addition, the in vitro synergistic therapeutic efficacy was further evaluated by a Calcein AM/PI co-staining test. Likewise, synergistic treatment induced the largest number of 4T1 cell death, as displayed by the red fluorescence of PI (Fig. 5c). These findings strongly agree with the CCK-8 results. It is worth noting that the current strategy of synergistic PDT/PTT and hypoxia-triggered chemotherapy shows obvious therapeutic effects in vitro even under the irradiation of a single wavelength near infrared laser with a very low power density (0.8 W cm^− 2^), which is far superior to previous reported studies (1–1.5 W cm^−2^) [44–46].

### In vivo biodistribution and metabolism of the APP NPs

**Fig. 6:**
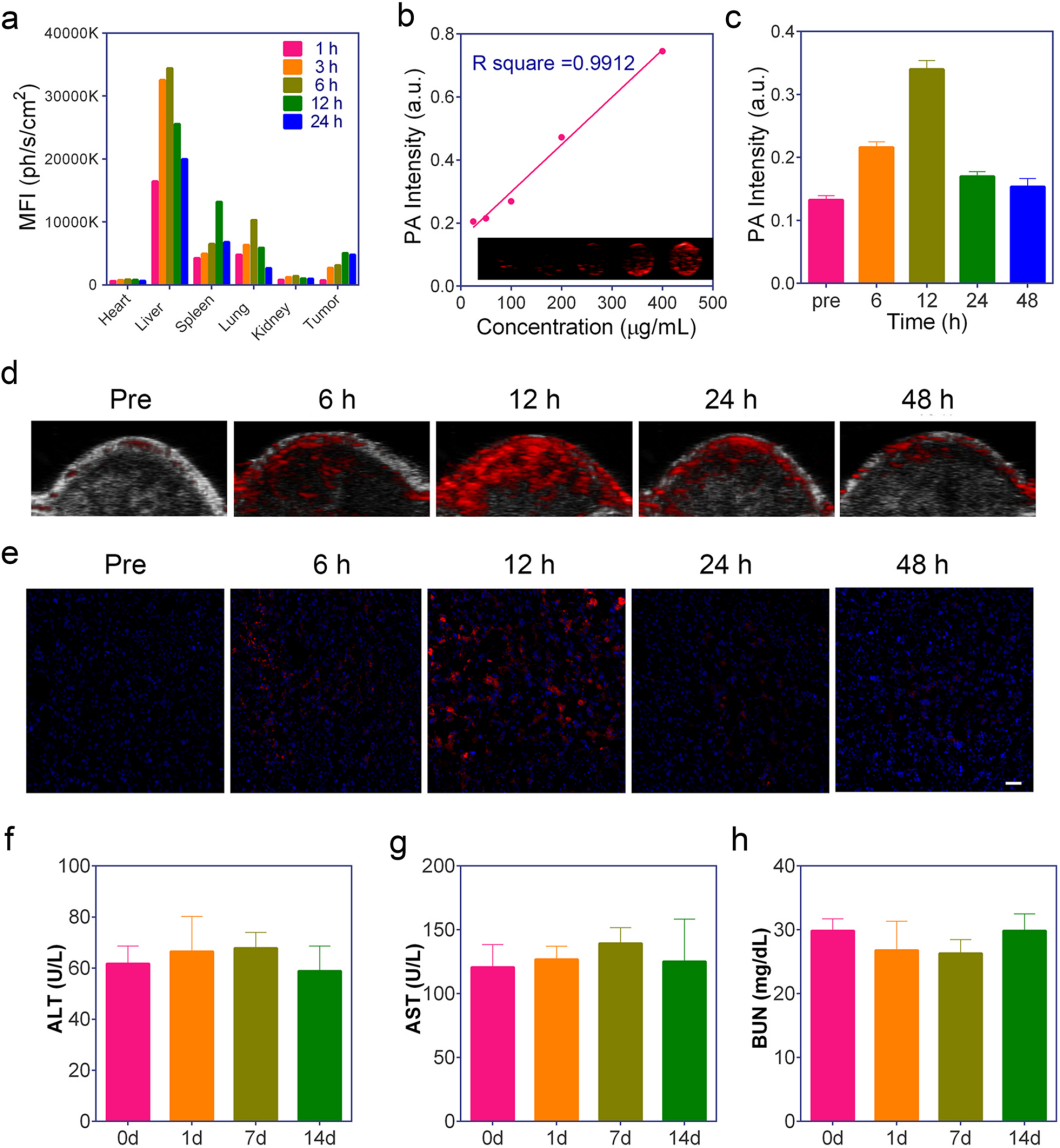
**a** Fluorescence intensities of main organs and tumors after injection of APP NPs (n = 3). **b** In vitro PA images and fitted relationship between PA signal values versus different concentrations of APP NPs (25, 50, 100, 200, 400 µg mL^−1^). **c** Quantitative PA values and (**d**) in vivo PA images of tumors after intravenous administration of APP NPs at different time intervals. **e** Ultrathin sections of tumor tissue at various time points postinjection of APP NPs detected by fluorescence microscopy (scale bar: 50 μm). **f**–**h** Biochemical indicators of liver and kidney functions, including aspartate aminotransferase (AST), alanine aminotransferase (ALT), and blood urea nitrogen (BUN), of mice postinjection of APP NPs for 0, 1, 7, and 14 days (n = 3)

The biodistribution and metabolism of nanoparticles are highly crucial for their biosafety and further applications [47]. The in vivo biodistribution of APP NPs in 4T1 tumor-bearing mice was investigated. Mice were intravenously administered APP NPs (10 mg kg^−1^), and ex vivo fluorescence images were obtained at various time points (1, 3, 6, 12, and 24 h), as demonstrate in Fig. 6a and Additional file 1: Fig. S14. A strong contrast was observed in the tumor tissue 12 h post-injection, indicating there was remarkable APP NPs accumulation inside the tumor, and the APP NPs mainly accumulated in the liver and spleen 24 h later, which was attributed to inevitable macrophage uptake in the reticuloendothelial systems. Further examination of ultrathin tissue sections (Fig. 6e) also supported the efficient accumulation of APP NPs at 12 h post-administration. In addition, the content of Zr element in blood was determined by ICP-MS, and the pharmacokinetics of APP NPs was determined by the content change of Zr element at different time points. As demonstrate in Additional file 1: Fig. S15, our APP NPs showed prolonged blood circulation up to 24 h, with a circulation halftime of 6.06 h. In summary, the evidence indicates that APP NPs have an advantageous tumor distribution profile, including a higher tumor uptake efficiency and longer retention time, and the degradation and clearance of APP NPs may be related to the strong affinity between Zr clusters in the MOFs and the plentiful phosphate in the biological environments [48].

### In vitro and in vivo PA imaging

Photoacoustic imaging is a rapidly growing hybrid biomedical imaging modality competent in mapping the absorption of laser in biological tissues via the PA effect [49, 50]. With full spectrum scanning of PA imaging from 680 to 910 nm, the PA signal intensity of APP NPs reached its strongest at an excitation wavelength of 690 nm, which could decrease the interference of hemoglobin (760 nm) and oxygenated hemoglobin (850 nm) [51]. As shown in Fig. 6b, PA signals strengthened linearly with increasing APP NPs concentration under an excitation wavelength of 690 nm, demonstrating that APP NPs could be a good contrast agent for PA imaging. Subsequently, the feasibility of applying APP NPs as PA imaging agents were evaluated in vivo. As presented in Fig. 6c, d, the PA signal enhanced over time and reached a peak at 12 h post-injection. In addition, this tendency also agreed with the quantitative analysis of PA signal intensity, showing that the APP NPs have a considerable PA imaging ability and significantly accumulate at the tumor site in vivo.

### In vivo biocompatibility studies

Biocompatibility is a very important factor for theranostic agents to be clinically relevant.[52] To investigate the biocompatibility and potential toxicity of APP NPs in vivo, healthy mice were monitored for two weeks after intravenous administration with a dose of 10 mg kg^− 1^ APP NPs. According to Fig. 6f–h and Additional file 1: Fig. S16, compared to that of the control group, APP NPs did not increase levels of different blood biochemical markers associated with normal hepatic and renal functions. In addition, H&E staining of the major organs of the APP NPs-treated mice exhibited no obvious pathological changes, such as inflammation, cell necrosis or apoptosis (Additional file 1: Fig. S17). Hence, these findings clearly demonstrate the high biosafety of the APP NPs.

### In vivo synergistic therapy

**Fig. 7:**
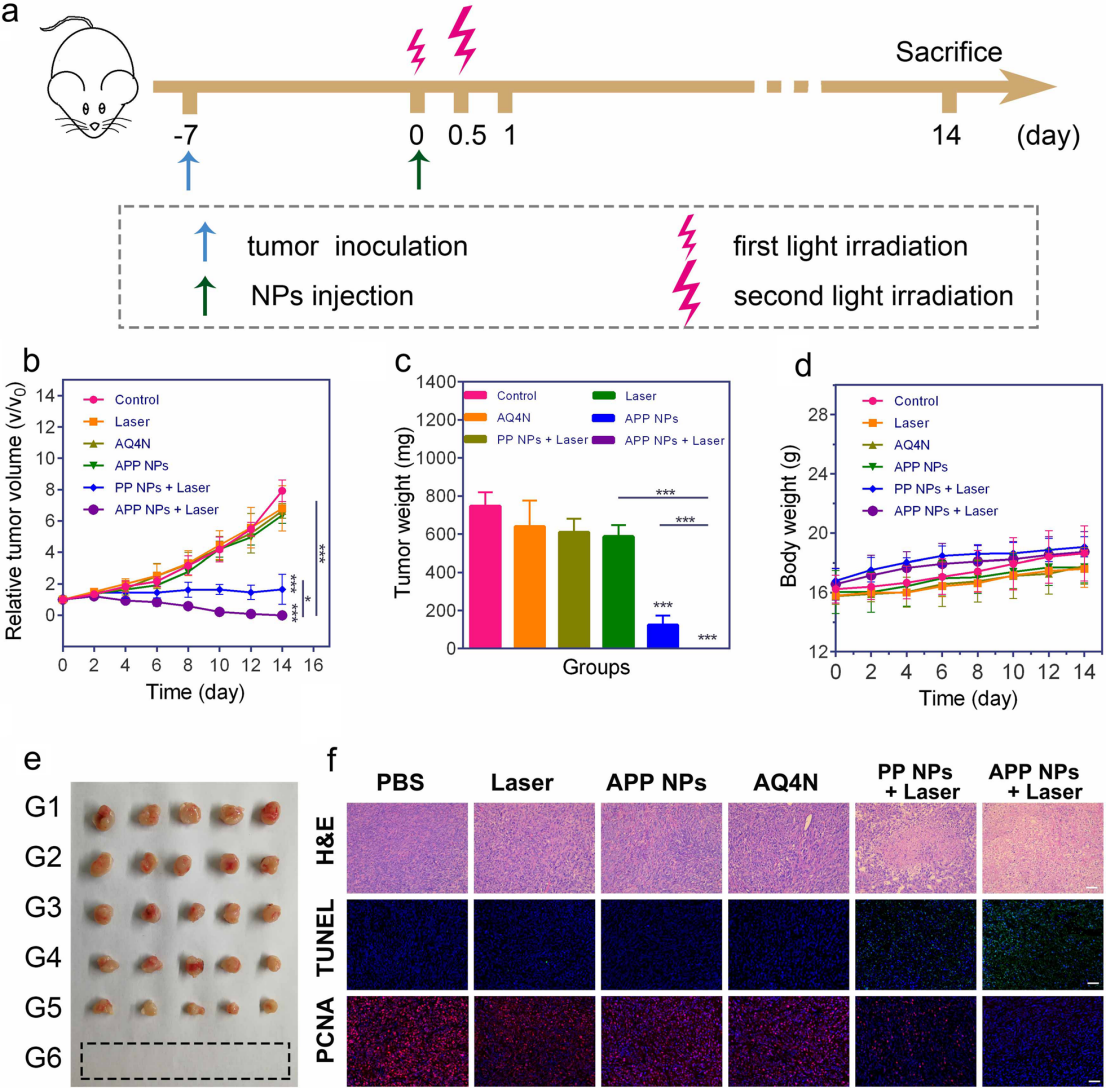
**a** Experimental procedure of antitumor study in vivo. **b** Tumor growth curves of mice after different treatments (n = 5). **c** Average weights of tumors at the end of the treatment (day 14). **d** Body weight of mice in each group. **e** Photographs of representative tumors dissected from various groups as indicated, G1: PBS, G2: Laser, G3: APP NPs, G4: AQ4N, G5: PP NPs + Laser, G6: APP NPs + Laser. **f** H&E and immunochemical staining of TUNEL and PCNA in tumor sections after different treatments, the scale bar is 50 μm. *p < 0.05, ***p < 0.01

Considering the favorable photochemotherapy effect of APP NPs in vitro, the therapeutic efficacy in vivo was assessed in 4T1 tumor-bearing mice. When the tumor volume reached approximately 80–100 mm^3^, all the mice were randomly divided into 6 groups (n = 5 per group), and i.v. injected with 200 µL PBS, free AQ4N, PP NPs or APP NPs. As shown in Fig. 7a, after intravenous administration, the tumor was first irradiated for 2 min with a 660 nm laser at 20 mW cm^−2^ for PCI-induced internalization; 12 h later, the groups that needed NIR irradiation were treated with a 660 nm laser. The tumor temperatures were monitored by an infrared thermographic imaging instrument. It was reported that both PDT and PTT could aggravate hypoxia levels in tumor microenvironments [53]. As shown in Additional file 1: Figs. S18 and S19, the temperature of the tumor in APP NPs + Laser group reached up to 57 °C within 5 min of 660 nm laser illumination (0.8 W cm^−2^). In contrast, the PBS + Laser group was not visibly heated, which suggested that the APP NPs could be used as an effective photothermal agent to locally heat tumors under a 660 nm laser. The body weights and tumor volumes of mice in each group were monitored for 14 days. Surprisingly, complete tumor elimination with no obvious recurrence was observed after treatment for 14 days in the APP NPs + Laser group (Fig. 7b, c, e), confirming the synergistic PTT/PDT and hypoxia-activated cascade photo-chemotherapy substantially enhanced therapeutic outcomes. The mice treated with PP NPs + Laser exhibited partial tumor growth inhibition of tumors due to the PTT/PDT effect, which might be caused by the existing resistance of cancer cells in the hypoxia region as a consequence of PDT/PTT. In contrast, tumors of the control group/laser-only group and the free AQ4N and APP NPs-only group grew progressively during the entire treatment period.

No body weight changes or potential damage or inflammation of main organs were observed after treated for 14 days (Fig. 7; Additional file 1: Fig. S20), suggesting that the APP NPs do not induce systemic toxicity. Moreover, hematoxylin & eosin (H&E), TdT-mediated dUTP nick-end labeling (TUNEL) and proliferating cell nuclear antigen (PCNA) staining of tumor slices further demonstrated that tumor tissues were destroyed more seriously by synergistic therapy compared to other treatment options (Fig. 7f), indicating that photo-chemotherapy featured the highest therapeutic efficacy and minimized side effects.

**Fig. 8:**
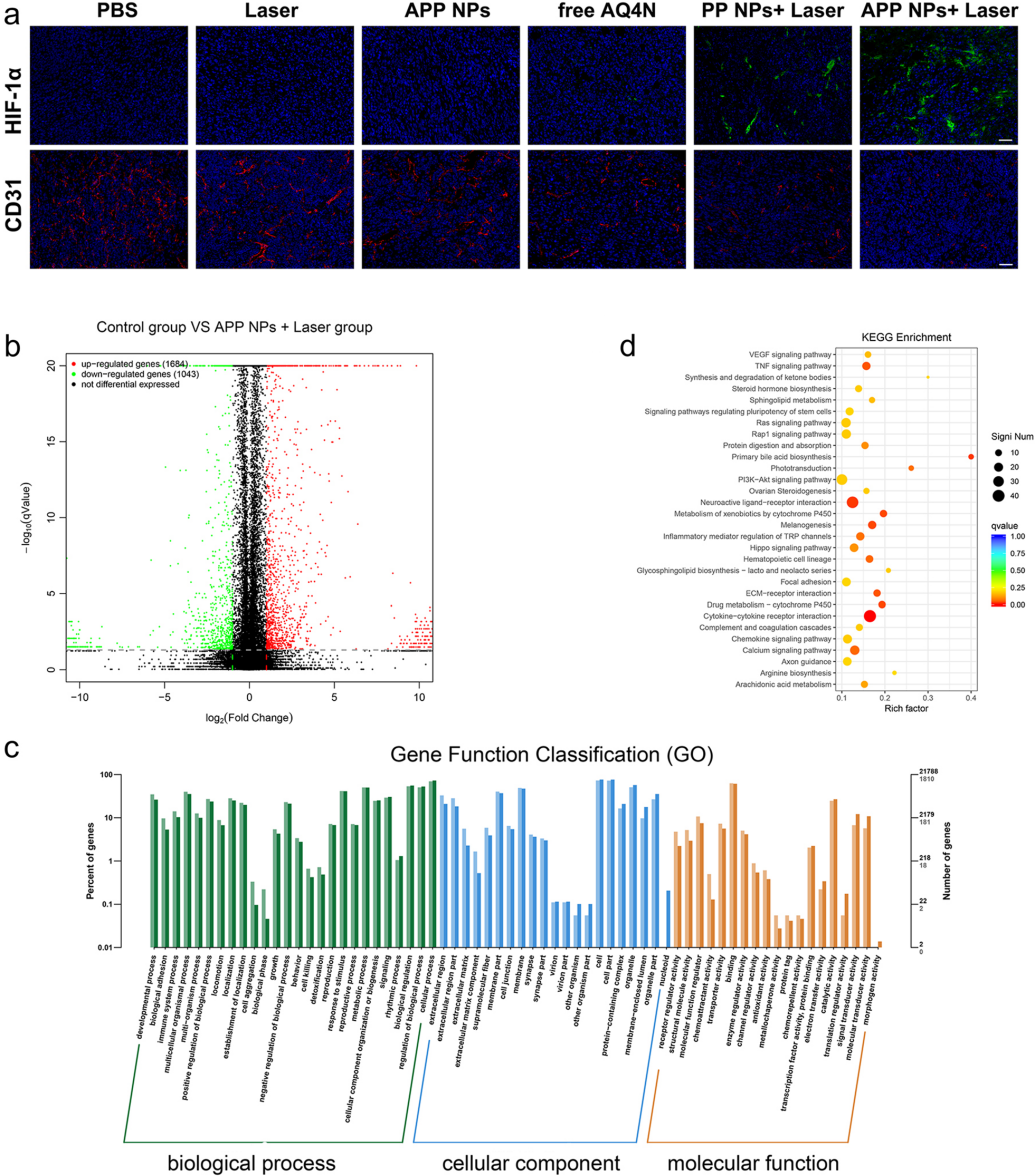
**a** Immunohistochemical analysis of HIF-1α and CD31 assays for tumors in various groups. The scale bar is 50 μm. **b** Volcano plots comparison of differentially expressed genes between control group and APP NPs + Laser group; red dots denote individually upregulated genes, and green dots denote individually downregulated genes. **c** GO terms of differentially expressed genes comparing the APP NPs + Laser group with the control group. **d** The top 30 overrepresented KEGG pathways comparing the control group with the APP NPs + Laser group

To further investigate PDT/PTT-enhanced hypoxia, hypoxia factor (HIF)-1α was detected as a probe for hypoxia detection using immunohistochemical analysis. As shown in Fig. 8a, a higher expression of HIF-1α was present in the PP NPs + Laser and APP NPs + Laser groups than in the other groups, suggesting significantly increased hypoxia caused by PP NPs and APP NPs during phototherapy. To further validate the possible reasons for increased hypoxia, platelet endothelial cell adhesion molecule-1 (CD31) staining was used to detect the blood vessel status of the tumor. Immunofluorescence staining of CD31 showed that the blood vasculature of the PP NPs + Laser and APP NPs + Laser groups was seriously damaged and angiogenesis was inhibited. Quantitative image analysis showed that the percentage of tumor blood vessel area in the APP NPs + Laser group was 0.47%, which was significantly lower than that in the control group (3.11%) (Additional file 1: Fig. S21). Obviously, during the process of PDT/PTT oxygen consumption, serious vascular damage occurred, which hindered the oxygen supply and aggravated hypoxia.

Next, we analyzed the mRNA profile of the negative control group with the APP NPs + Laser group by RNA sequencing to investigate the potential therapeutic mechanism of APP NPs under 660 nm laser irradiation. Differential gene expression was assessed by expression levels. Consequently, 2727 genes were differentially expressed when comparing the APP NPs + Laser group with the control group (P-adjust < 0.05), which included 1684 upregulated genes and 1043 downregulated genes (Fig. 8b). The gene ontology (GO) enrichment classification details were displayed in Fig. 8c. Two molecular function terms were significantly enriched. Kyoto Encyclopedia of Genes and Genomes (KEGG) pathway enrichment analysis revealed the pathways involved in differential gene expression, and it was conducted to acquire a better understanding of how APP NPs acted on tumor cells by photoinduced PDT/PTT combined chemotherapy. Notably, “Metabolism of xenobiotics by cytochrome P450” and “Drug metabolism-cytochrome P450” were significantly enriched after combined treatment (Fig. 8d). Interestingly, in addition to hypoxia, AQ4N activation is dependent on activation by cytochrome P450 [54]. These results demonstrated that APP NPs treatment and subsequent NIR laser irradiation could effectively active AQ4N into toxic AQ4.

## Conclusion

In conclusion, we successfully synthesized a multifunctional nanoplatform for PA imaging guided, efficient intracellular delivery and synergistic photochemotherapy under dual-stage irradiation with a NIR laser. The nanosystem was constructed by a simple, one-pot process. The synthesized APP NPs exhibited biocompatibility, low toxicity, and high stability both in vitro and in vivo. As expected, the sequential activation of NIR-induced phototherapy and hypoxia-induced bioreductive chemotherapy have been demonstrated to completely eradicate the cell/tumor both in vitro and in vivo, which could overcome the drawback of traditional PDT/PTT. This work thus represents a versatile strategy to construct a single wavelength NIR laser-induced PDT/PTT and hypoxia-activated chemotherapy nanoplatform and will be promising for the development of multistimuli-responsive nanosystems with programmable functions for precise cancer therapy.

## Methods

### Synthesis of PCN-224, AQ4N@PCN-224, AQ4N@PCN-224@PDA

For the synthesis of PCN-224: first, TCPP (100 mg), benzoic acid (2.8 g) and ZrOCl_2_·8H_2_O (300 mg) were added in DMF (100 mL) and stirred at 90 °C for 5 h. Then, the precipitate was collected by centrifugation (8000 g, 10 min) and washed with DMF three times.

1 mg AQ4N was dissolved in 1mL deionized water, mixed with PCN-224 solutions (1 mg mL^−1^), and then incubated at 37 °C for 12 h under magnetic stirring. The mixtures were centrifuged (8000 g, 10 min) and washed with three times to obtain AQ4N@PCN-224 (AP NPs) nanoparticles.

AQ4N@PCN224@PDA (APP NPs) were synthesized using the prepared AP NPs as the template. Briefly, dried AQ4N@PCN-224 (10 mg) was added in a water-ethanol mixture (14 mL, 4:3, v: v). Next, dopamine hydrochloride (8 mg) was added into the solution under magnetic stirring. After stirring for 5 min, 20 mL of aqueous solution of Tris (hydroxymethyl) aminomethane (25 mM) was added and the mixture was stirred for 24 h in the dark. Then, the resulting solution was centrifugation (8000*g*, 10 min) and washed. Finally, APP NPs were obtained. The preparation of PCN224@PDA (PP NPs) was in the same way as APP NPs, expect that AQ4N were not added.

### Photothermal-triggered AQ4N release behavior

To investigate the in vitro AQ4N release from the nanoparticles, 5 mL of APP NPs (200 µg mL^−1^, equivalent to the concentration of AQ4N) was suspended in 20 mL of PBS solution (37 °C, pH 7.4) under gentle stirring. Triggered by 660 nm laser (0.8 W cm^−2^), the released free AQ4N was determined spectrophotometrically every 2 min.

### Photothermal capability in vitro

APP NP aqueous solutions at various concentrations (50, 100, 200, 300, 400 µg mL^−1^) were exposed to a 660 nm laser (0.8 W cm^−2^), and monitored by an infrared thermal imaging camera. Water was used as the control. In addition, the PTT stability of the APP NPs (50 µg mL^−1^) was detected. Typically, the APP NPs solution was irradiated with NIR laser (660 nm, 0.8 W cm^−2^) for 600 s in a quartz cuvette and then cooled to indoor temperature. The photothermal conversion efficiency () of APPs was measured under 660 nm laser irradiation (Additional file 1).

### Singlet oxygen generation detection in vitro

SOSG was used as a singlet oxygen indicator. Typically, 0.2 mL of APP NPs (50 µg mL^−1^) or PCN-224 (50 µg mL^−1^) was added to 96-well plates, after which SOSG (5 µM) was added, and irradiated with an NIR laser (660 nm, 100 mW cm^−2^). Equal deionized water supplemented with SOSG was used as a control. The fluorescence intensity of SOSG was detected by a multimode reader (excitation wavelength: 494 nm).

### PCI-induced internalization measurement

4T1 cells were inoculated into CLSM-exclusive culture dish for 24 h. Then, APP NPs (50 µg mL^−1^) was co-cultured with the cells for 1 h. Subsequently, the cells were treated with 660 nm laser (20 mW cm^−2^) for 2 min per dish. After co-cultured for another 2 h, cells were treated with DAPI for nuclear staining and observed with CLSM. In addition, PCI-induced internalization was further analyzed by flow cytometry. Cells in six-well plates were treated using the same method and analyzed by flow cytometry.

### Detection of cellular ROS generation

The 4T1 cells were treated with APP NPs (PCN-224 = 50 µg mL^−1^) and 1 mL of PCN-224 (50 µg mL^−1^). Then, the cells were co-cultured with DCFH-DA (10 µM) for 60 min; subsequently, administered with 660 nm laser (100 mW cm^−2^) for 5 min. Cells without any treatments were used as a control. The ROS production ability of APP NPs was analyzed by CLSM and flow cytometry.

### In vitro toxicity evaluation

HUVECs and 4T1 breast cancer cells were treated with various concentrations (0, 10, 25, 50, 100, 200 µg mL^−1^) of APP NP aqueous solutions for 24 h. The CCK-8 method was used to assess the cytotoxicity of APP NPs in vitro.

### Synergistic antitumor efficacy of APP NPs in vitro

The relative cell viability of 4T1 cells was used to assess the therapeutic efficacy of APP NPs. The 4T1 cells were seeded in 96-well plates for 24 h. Then, treated with APP NPs (50 µg mL^−1^) or PBS for 1 h and received the first 660 nm laser irradiation (2 min at 20 mW cm^−2^). After co-incubation for another 3 h, the 4T1 cells received various treatments, including Laser, free AQ4N, APP NPs + Laser, PP NPs + Laser + hypoxia, and APP NPs + Laser + hypoxia. The 4T1 cells were irradiated with a 660 nm laser (5 min, 0.8 W cm^−2^). Finally, cell viability was evaluated by the CCK-8 assay. Similarly, a living/dead cell staining assay against 4T1 cells was performed by CLSM. The apoptosis of 4T1 cells induced by APP NPs was detected by an Annexin V-FITC/PI staining assay. Briefly, 4T1 cells were seeded in 6-well plates for 24 h incubation, received various treatments as mentioned above. Finally, cell viability was detected by flow cytometry.

### Biodistribution and metabolism of the APP NPs

To investigate the biodistribution of APP NPs, tumor-bearing mice were intravenously injected with APP NPs (200 µL, 10 mg kg^−1^), sacrificed and dissected at 1, 3, 6, 12 and 24 h. The corresponding fluorescence images of tumors and major organs (heart, spleen, liver, lung, kidney) were obtained, and the relative fluorescence intensity was measured. To further investigate the metabolism of the APP NPs, after intravenous injection with APP NPs (200 µL, 10 mg kg^−1^), mice were sacrificed and dissected at 0, 6, 12, 24, and 48 h to collect tumors. Then, the tissues were dissected to obtain ultrathin sections and were stained with DAPI for fluorescence imaging.

### In vitro and in vivo PA imaging

In vitro PA imaging, various concentrations of obtained APP NPs (25, 50, 100, 200, 400 µg mL^−1^) were dispersed in ultrapure water and scanned for PA signal detection and to assess the linearity between PA signals and APP NPs concentrations.

In vivo PA imaging were performed on 4T1 tumor-bearing mice by intravenous injected of APP NPs (200 µL, 10 mg kg^−1^). After injection, the tumors were carried out with 660 nm NIR light (20 mW cm^−2^, 2 min) for PCI-induced internalization. The PA images were simultaneously recorded at determined time points (0, 6, 12, 24, and 48 h).

### In vivo light/hypoxia programmable triggered photochemotherapy

To evaluate the in vivo photochemotherapy efficacy of APP NPs, when the tumor volume increased to 80–100 mm^3^, mice were randomly divided into six groups (n = 5 each group): the control, Laser, AQ4N, APP NPs, PP NPs + Laser, and APP NPs + Laser groups. Two hundred microliters of PBS, free AQ4N and various nano-formulations (10 mg kg^− 1^) were injected into mice by tail intravenous injection. The tumors were irradiated with 660 nm NIR light (20 mW cm^−2^, 2 min) for PCI-induced internalization after injection in all groups. 12 h later, the laser group was irradiated with a 660 nm NIR laser (0.8 W cm^−2^, 5 min). Simultaneously, the temperature of tumor was recorded by an infrared thermal imaging camera. Finally, all tumors and major organs were harvested for further histological analysis. For the APP NPs + Laser group, because the tumors were eliminated entirely, another group was added for histological analysis.

## Supplementary Information

**Additional file 1:** Additional Figures.

## Data Availability

All data generated or analyzed during this study are included in this article.
