# Supplementary material for: Photochemically-driven highly efficient intracellular delivery and light/hypoxia programmable triggered cancer photo-chemotherapy
Source: J Nanobiotechnology. 2023 Jan 12;21:11. doi: 10.1186/s12951-023-01774-w (PMC9835365; doi:10.1186/s12951-023-01774-w)
Supplement: Supplementary file 1 — Additional file 1: Additional Figures. [file 12951_2023_1774_MOESM1_ESM.docx]

**Photochemically-Driven Highly Efficient Intracellular Delivery and Light/Hypoxia Programmable Triggered Cancer Photo-Chemotherapy**

*Wei Zhang^1^, Cuncheng Zhang^2^, Chao Yang^3^, Xingyue Wang^1^, Weiwei Liu^1^, Mi Yang^1^, Yang Cao^1^, and Haitao Ran^1^**

^1^ Chongqing Key Laboratory of Ultrasound Molecular Imaging & Department of Ultrasound, Second Affiliated Hospital of Chongqing Medical University, No. 74 Linjiang Rd, Yuzhong District, Chongqing, 400010, China.

^2^ Department of Ultrasound, Chongqing General Hospital, No. 104, Pipashan Main Street, Yuzhong District, Chongqing, 40013, China.

^3^ Department of Radiology, Chongqing General Hospital, No. 104, Pipashan Main Street, Yuzhong District, Chongqing, 40013, China.

Correspondence should be directed to Haitao Ran at the following address:

Institution and address: Chongqing Key Laboratory of Ultrasound Molecular Imaging & Department of Ultrasound, Second Affiliated Hospital of Chongqing Medical University, No. 74 Linjiang Rd, Yuzhong District, Chongqing, 400010, PR China.

Email: ranhaitao@cqmu.edu.cn

**Materials**

Tetrakis (4-carboxyphenyl) porphyrin (TCPP) was purchased from Tokyo Chemical Industry CO., Ltd. Chloride octahydrate (ZrOCl_2_·8H_2_O), benzoic acid (BA) and N,N’-dimethylformamide (DMF) were obtained from Adama-Beta Co., Ltd. Dopamine hydrochloride was purchased from Aladdin. 2’,7’-Dichlorofluorescin diacetate (DCFH-DA), calcein-AM, PI and Cell Counting Kit-8 were purchased from Dojindo (Japan). AQ4N were purchased from Abcam. All commercial chemicals were used as received without further purification unless specified otherwise. All cell culture-relevant reagents were obtained from Beyotime Institute of Biotechnology (China).

**Characteristics of APP NPs**

Transmission electron microscopy (TEM, HIitachi h7600, Japan) and scanning electron microscopy (SEM, Hitachi S-3400N, Japan) were used to perform the morphological characterization. High-angle annular dark field (HAADF) STEM and energy-dispersive X-ray (EDX) analyses were performed on a ThermoFisher Scientific Talos F200S field-emission transmission electron microscope. A Malvern Zetasizer Nano ZS90 analyzer was used to determine the size distribution and zeta potential analysis at room temperature. The UV–Vis absorption spectra were recorded on a UV–Vis spectrophotometry lambada 950 (PerkinElmer). The ESR characterization was performed on Bruker EMX Electron paramagnetic Resonance (EPR) Spectrometer.

**Photothermal conversion efficiency**

The photothermal conversion efficiency (𝜂) of APPs was calculated according to following equations:

𝜂 = $hS (T_{\mathrm{Max}}-T_{\mathrm{Surr}})-Q\mathrm{Dis}$ $\frac{hS (T_{Max}-T_{Surr})-QDis}{I (1-{10}^{-A660})}$ $\frac{hS (T_{\mathrm{Max}}-T_{\mathrm{Surr}})-Q\mathrm{Dis}}{I (1-{10}^{-A808})}$

*𝜏_𝑠_ =* $\frac{M_{D}C_{D}}{hS}$

Where $h$ denotes the coefficient of heat transfer,$S$ is container’s surface area, and the value of $hS$ calculated from the Figure S, $(T_{Max}-T_{Surr})$ represents teperature change of the APP NPs auueous solution at the maximal steady-stage temperature, and $I$ is laser power. *A_660_* is the absorbance intensity of APP NPs at 660 nm and $QDis$ express the heat accociated with light absorption by the solvent. *𝜏_𝑠_* represents the time constant of sample-system. *C_D_* and *M_D_* are the heat capacity and mass of the solvent H_2_O, respectively.

**Drug Loading Content**

The drug loading content of AQ4N in APP NPs was measured by a UV–vis spectrophotometer at 610 nm and calculated by a standard curve. AQ4N encapsulation efficiency = (M_t_-M_f_)/M_t_, AQ4N loading efficiency = (M_t_-M_f_)/M_NP_. where M_t_ refers to the weight of AQ4N fed, M_f_ refers to the weight of unencapsulated AQ4N, and M_NP_ refers to the weight of nanoparticles.

**Animals and Tumor Xenograft Model**

Female Balb/c mice at 6-8 weeks old were obtained from the Animal Center of Chongqing Medical University. To establish the 4T1 tumor model, 4 × 10^5^ 4T1 breast cancer cells were subcutaneously injected into the breast pads of each mouse. Animals received care in accordance with the guidance suggestions for the Care and Use of Laboratory Animals. All animal experiments were approved by the Animal Ethics Committee of Chongqing Medical University.

**Blood Biochemistry and Routine Examination**

Healthy Balb/c mice were intravenously injected with APP NPs (200 μL, 10 mg kg^-1^). At 1, 7 and 14 days postinjection, the mice were sacrificed, and blood samples and main organs were collected for blood analysis, biochemistry assay, and H&E staining analysis.

**Statistical Analysis**

All data reported are mean result ± SD. A *t test* was applied to detect the differences between groups: **p* < 0.05, ****p* < 0.01.


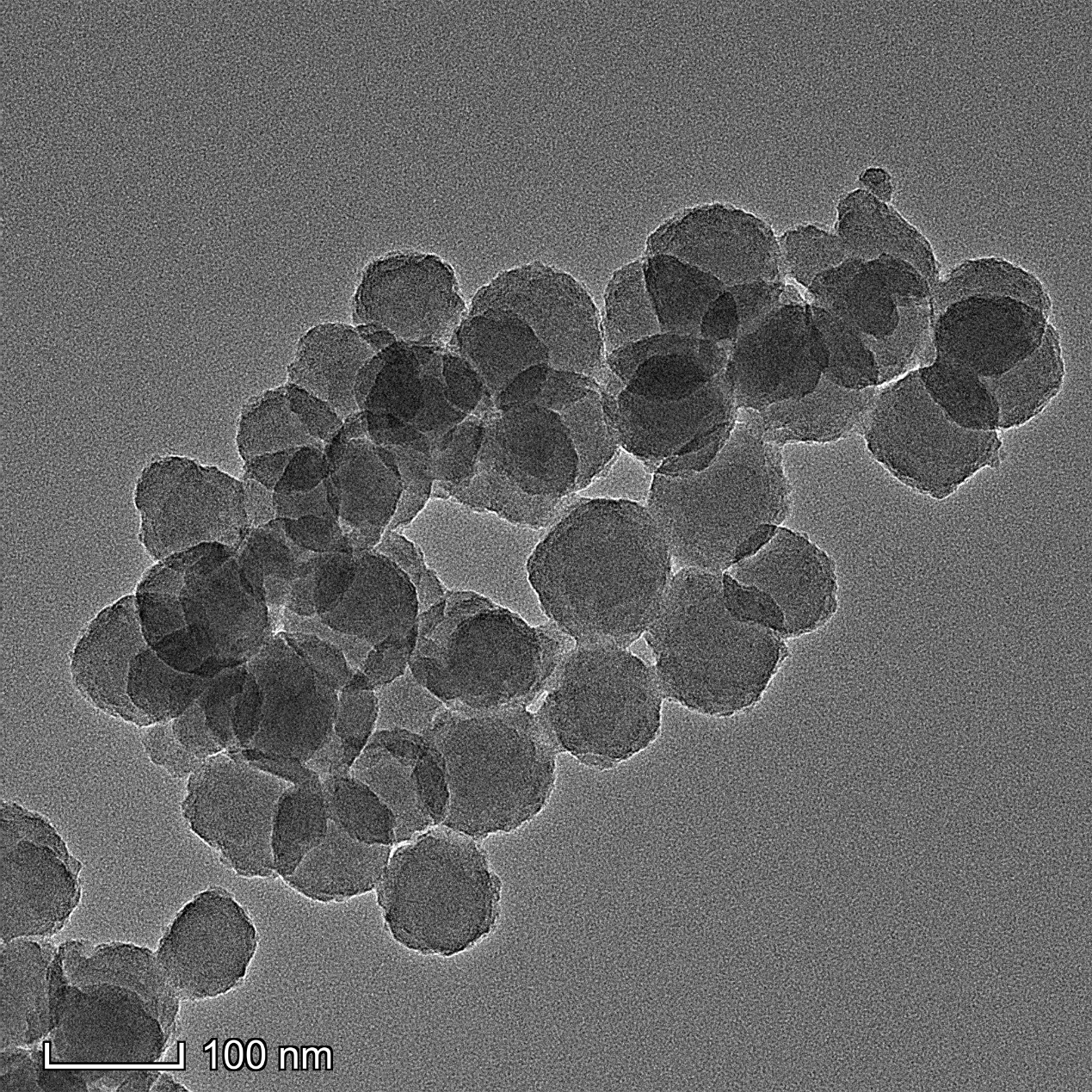


Figure S1 TEM images of PCN-224


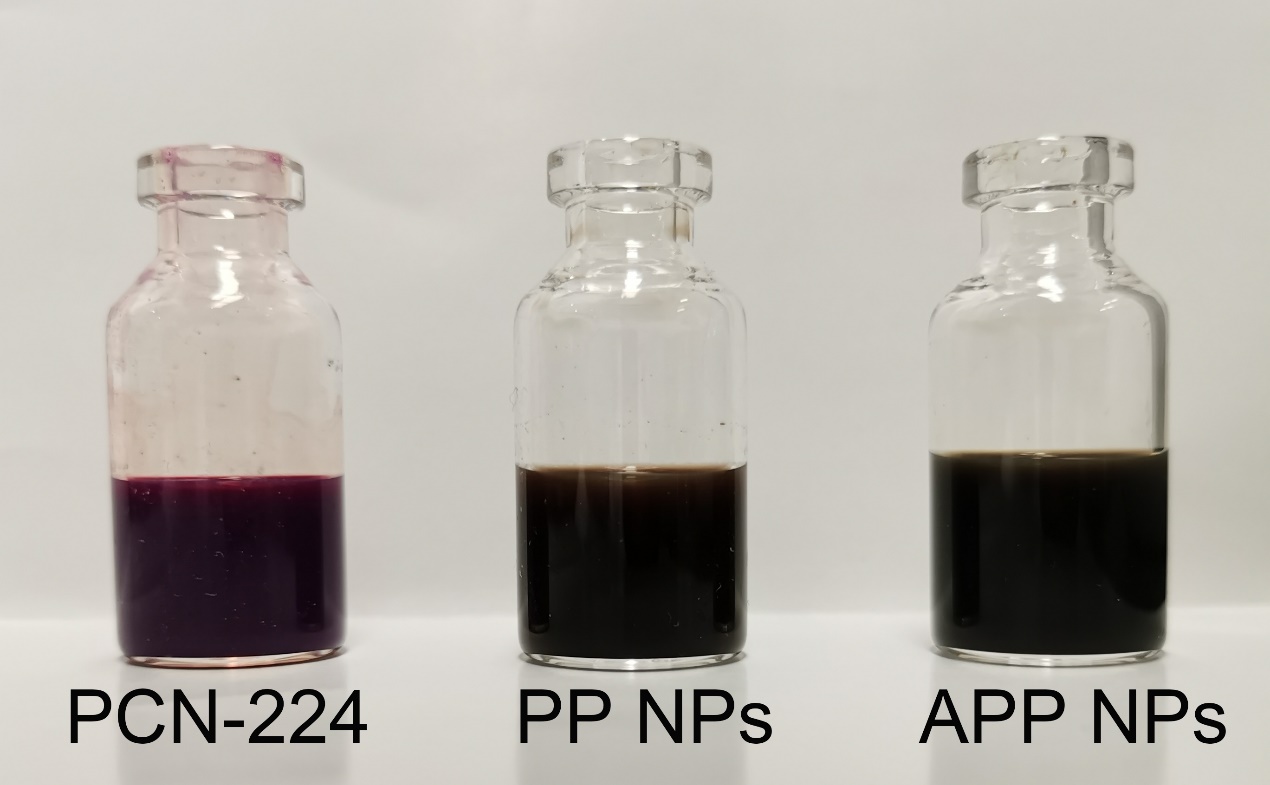


Figure S2 Photographic images of PCN-224, PP NPs, and APP NPs.


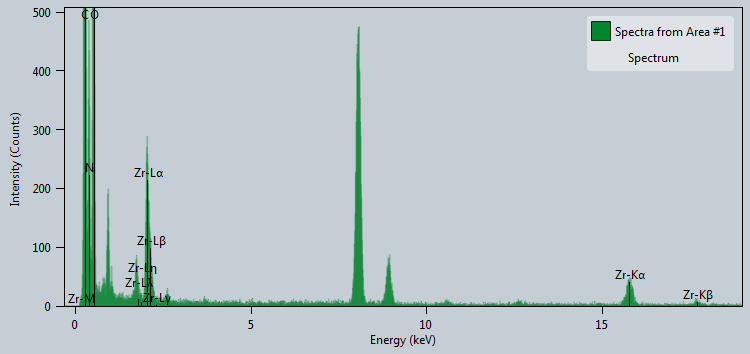


Figure S3 EDS spectrum of APP NPs


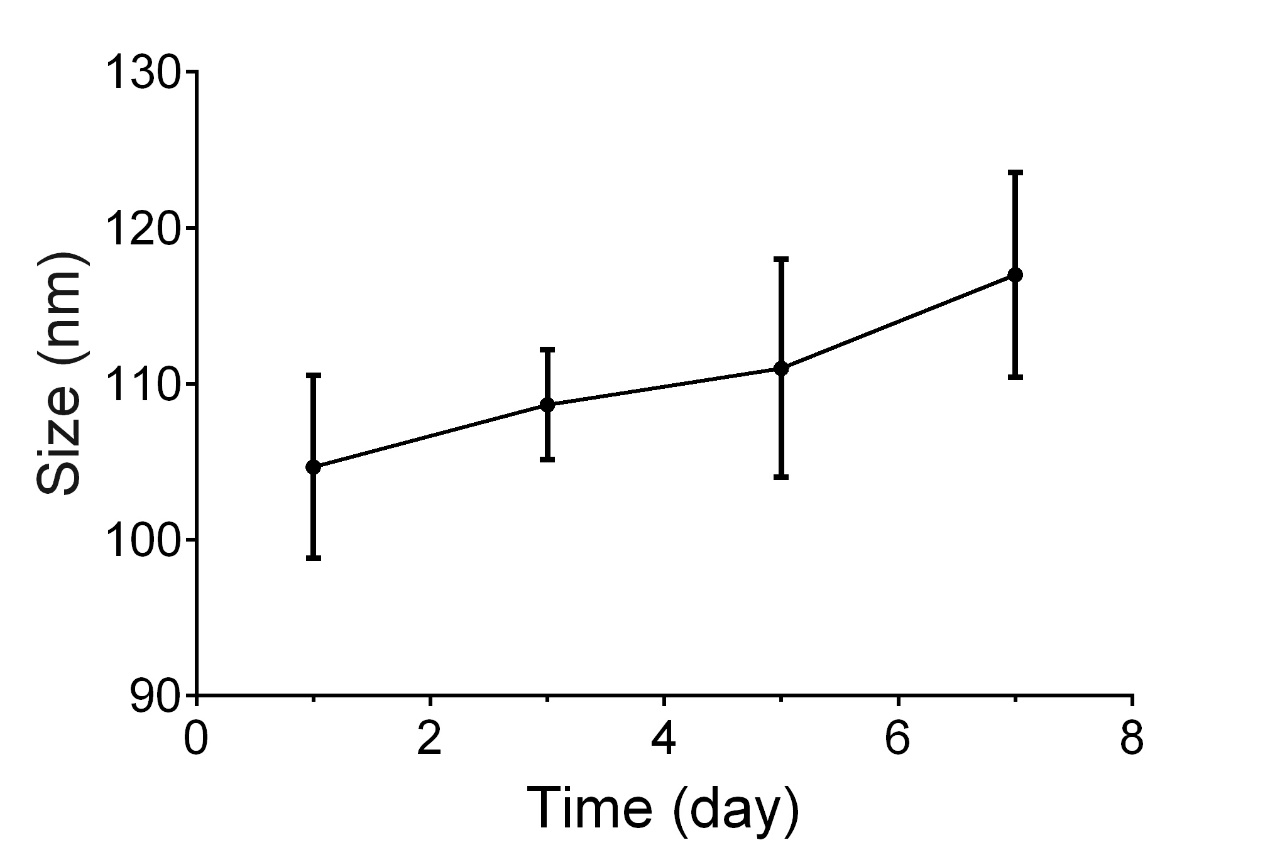


Figure S4 The size distribution with prolonged time duration.


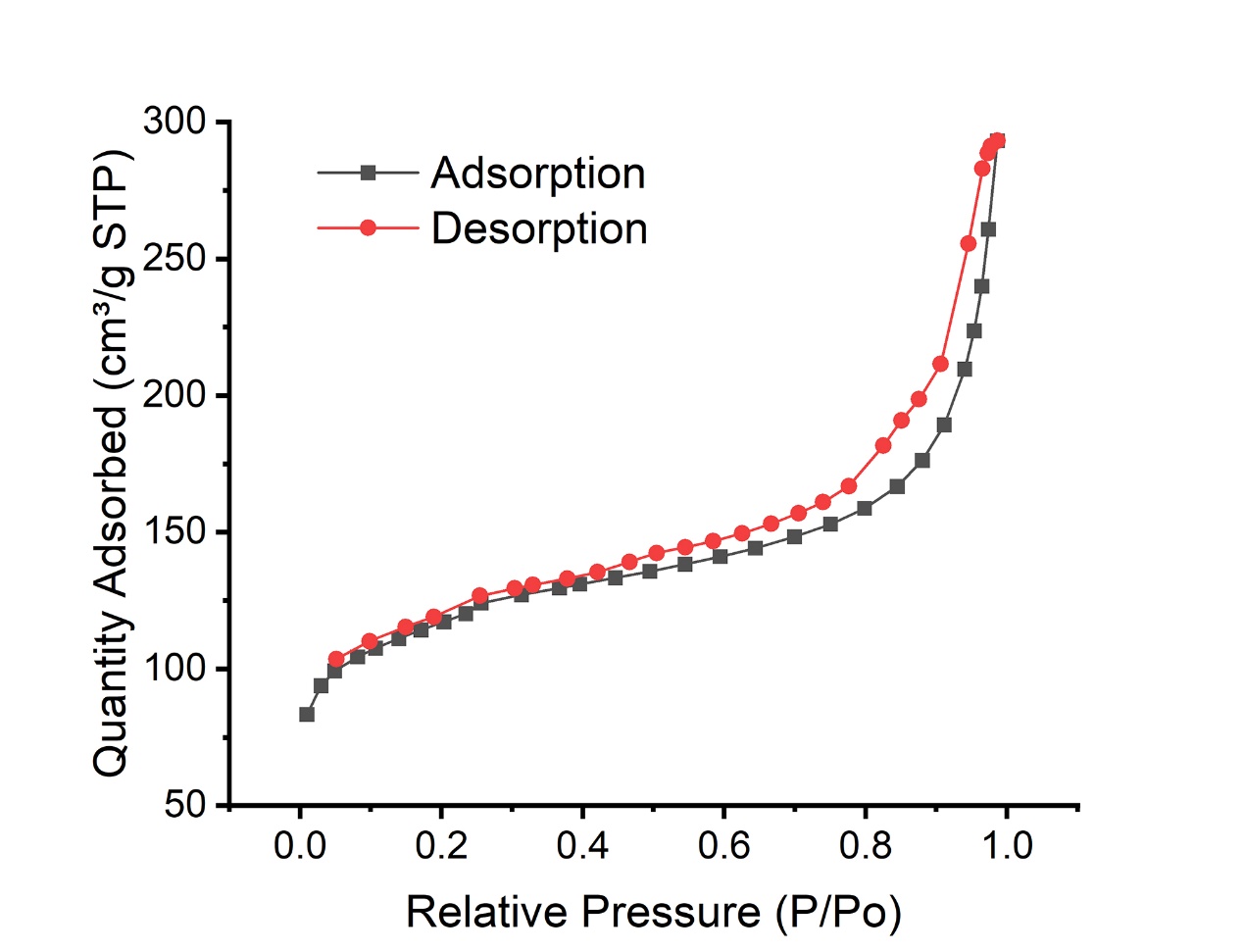


Figure S5 N_2_ adsorption-desorption isotherms of PCN-224


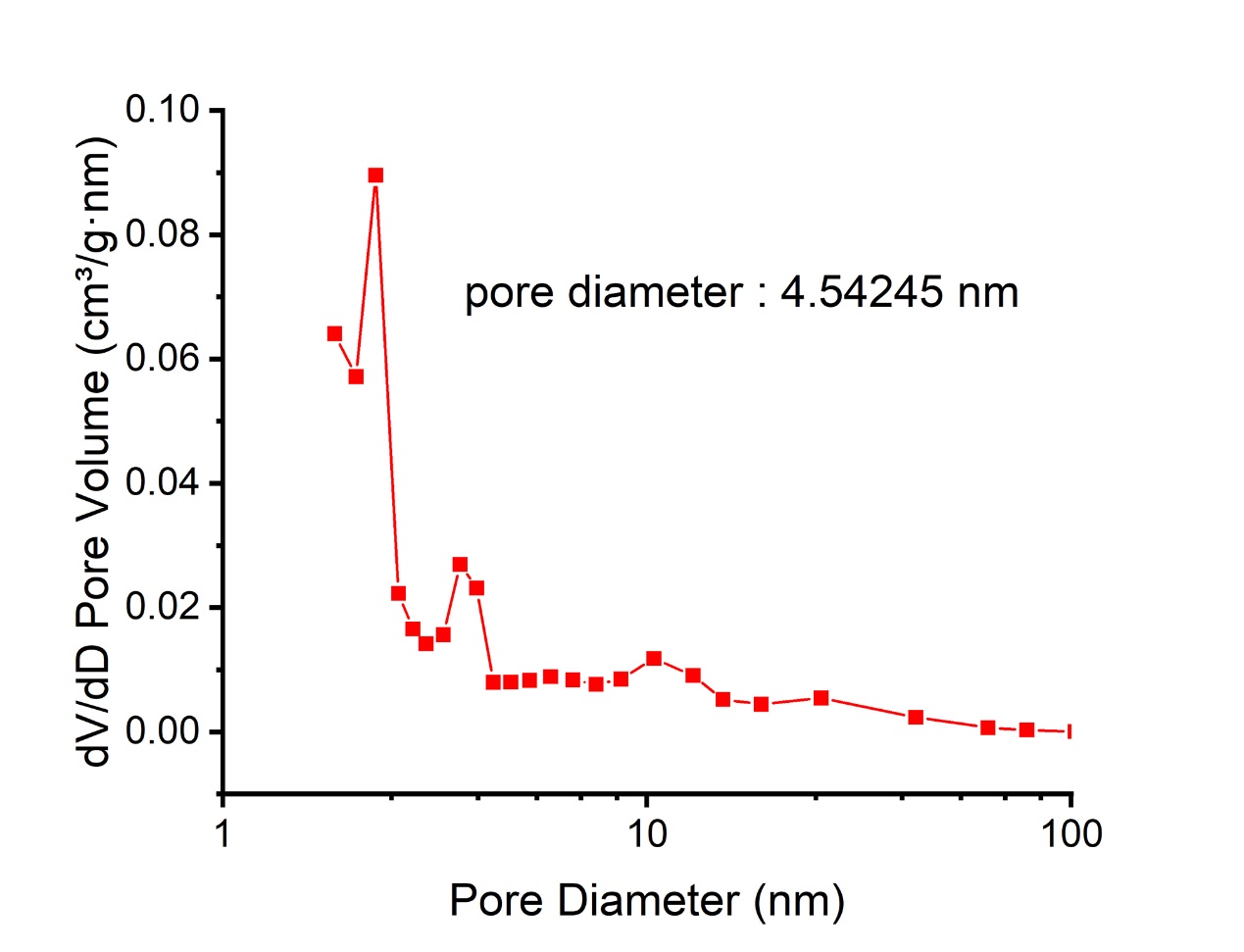


Figure S6 Pore-size distribution of PCN-224


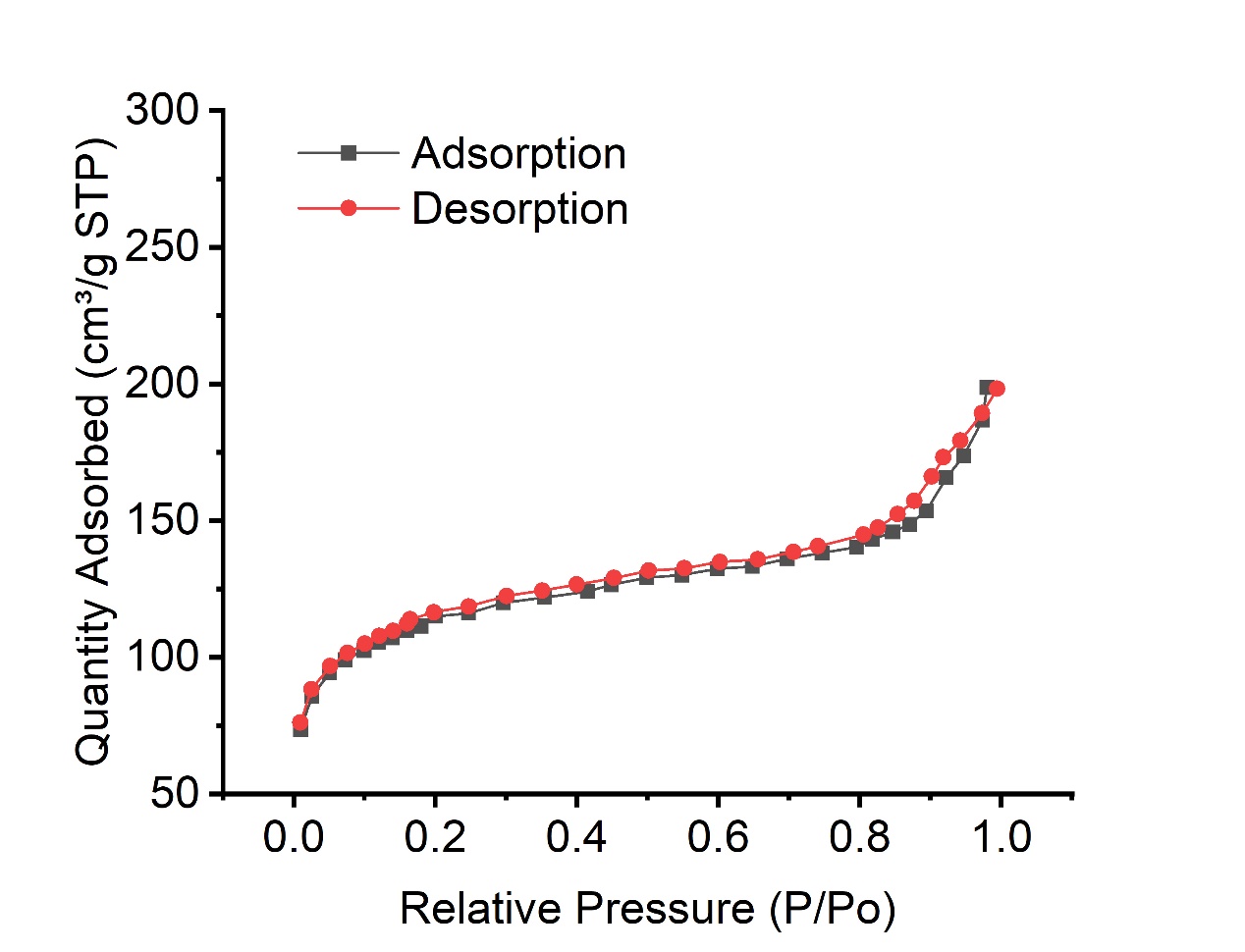


Figure S7 N_2_ adsorption-desorption isotherms of PCN-224@PDA


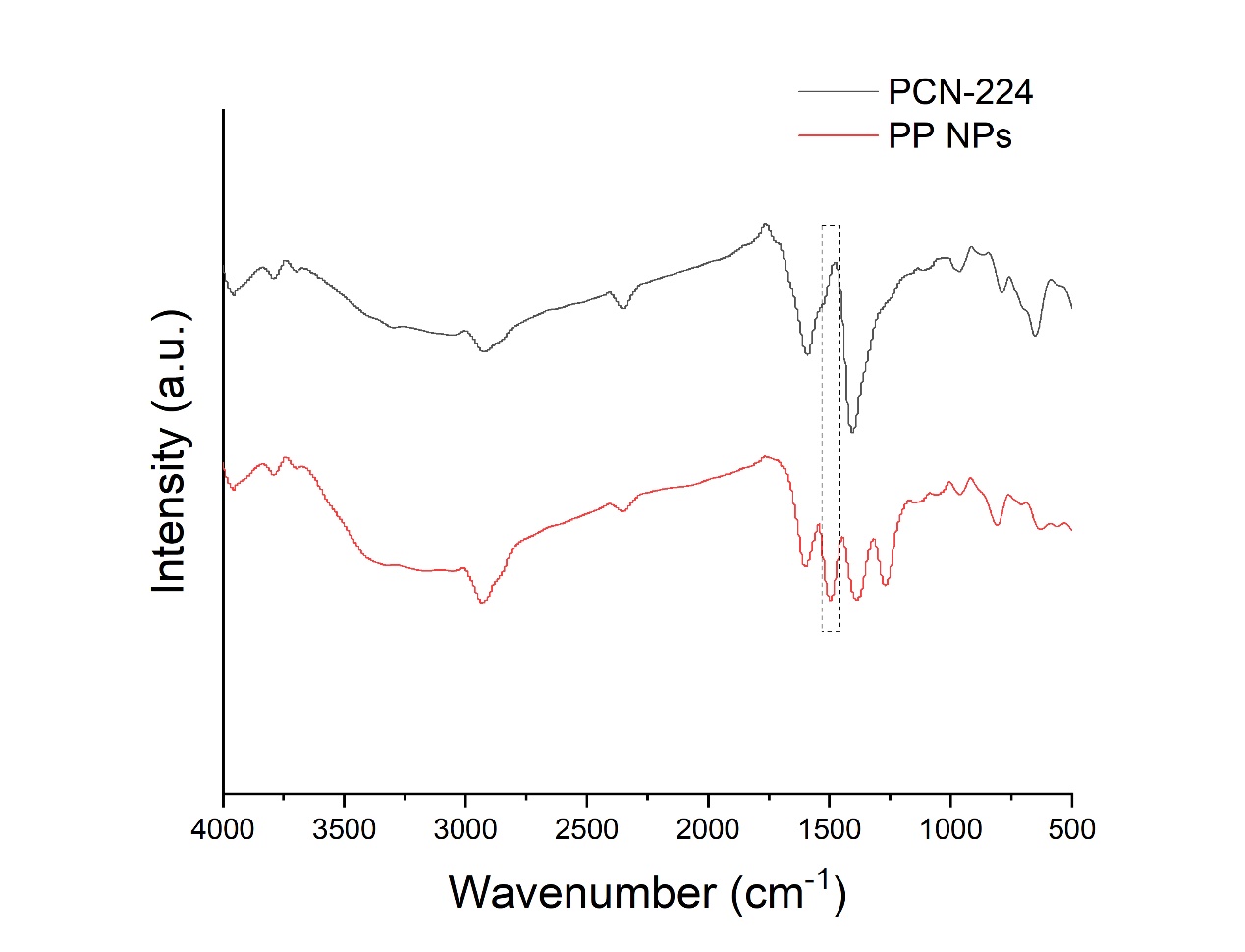


Figure S8 The FTIR spectra of PCN-224 and PP NPs. And after the PDA coating process, the peaks corresponding to the methylene in PDA appeared at about 1490 cm^-1^ in the spectrum of PP NPs.


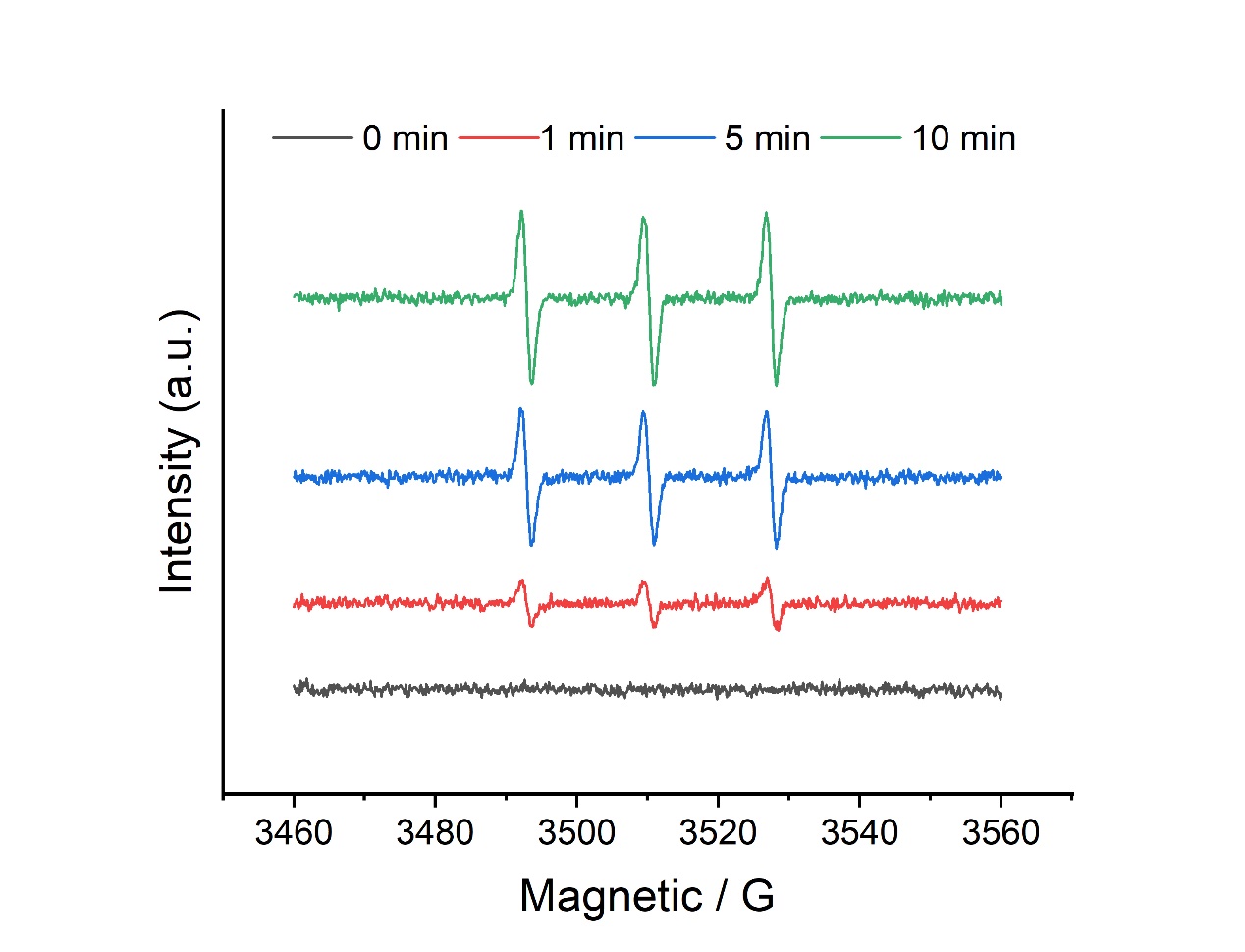


Figure S9 ESR spectra of ^1^O2 trapped by TEMP in APP NPs dispersions upon laser irradiation for prolonged durations.


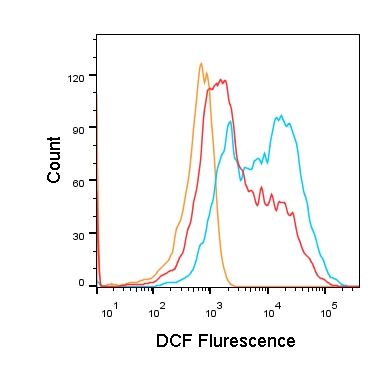


Figure S10 Intracellular ROS detection after treated with TCPP +light (orange), PCN-224+light (red), APP NPs +light (blue)


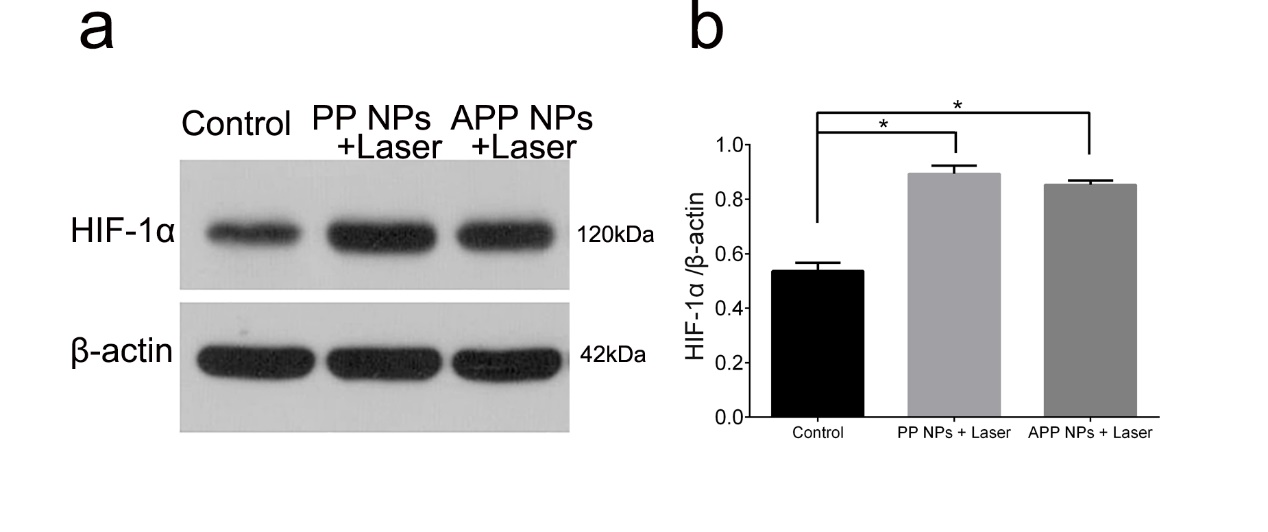


Figure S11 a) HIF-α protein expression levels of 4T1 cells after different treatments by western blot assay and b) corresponding statistical analysis result. **p*<0.05.


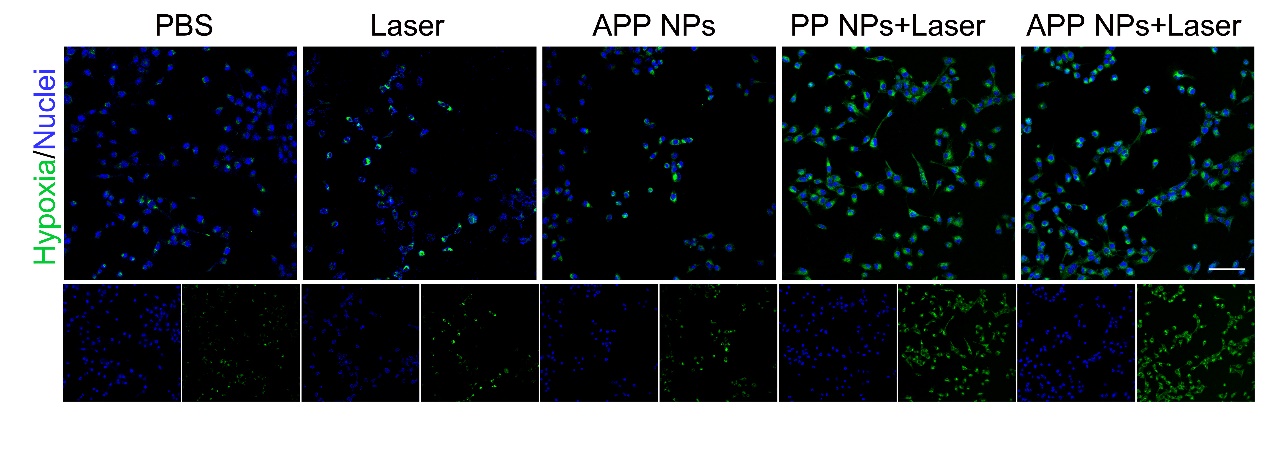


Figure S12 CLSM images of cells stain with hypoxia probes Hypoxyprobe (green). Scale bar is 50 μm.


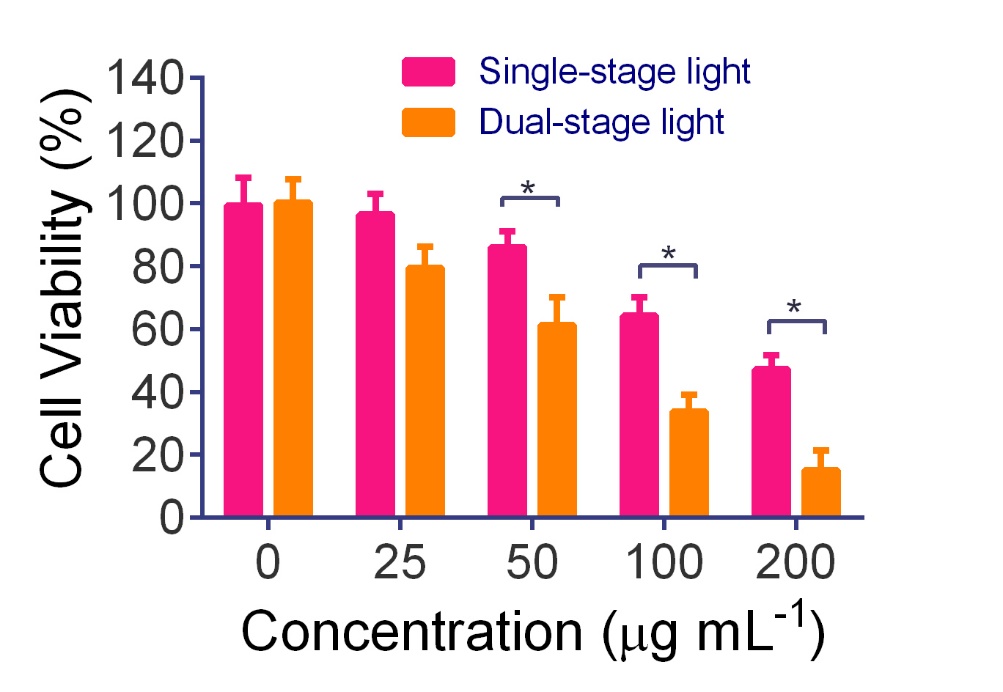


Figure S13 Cell viability of 4T1 cells upon single/dual-stage light irradiation after incubation with varying doses of APP NPs (n=5, *p < 0.05).


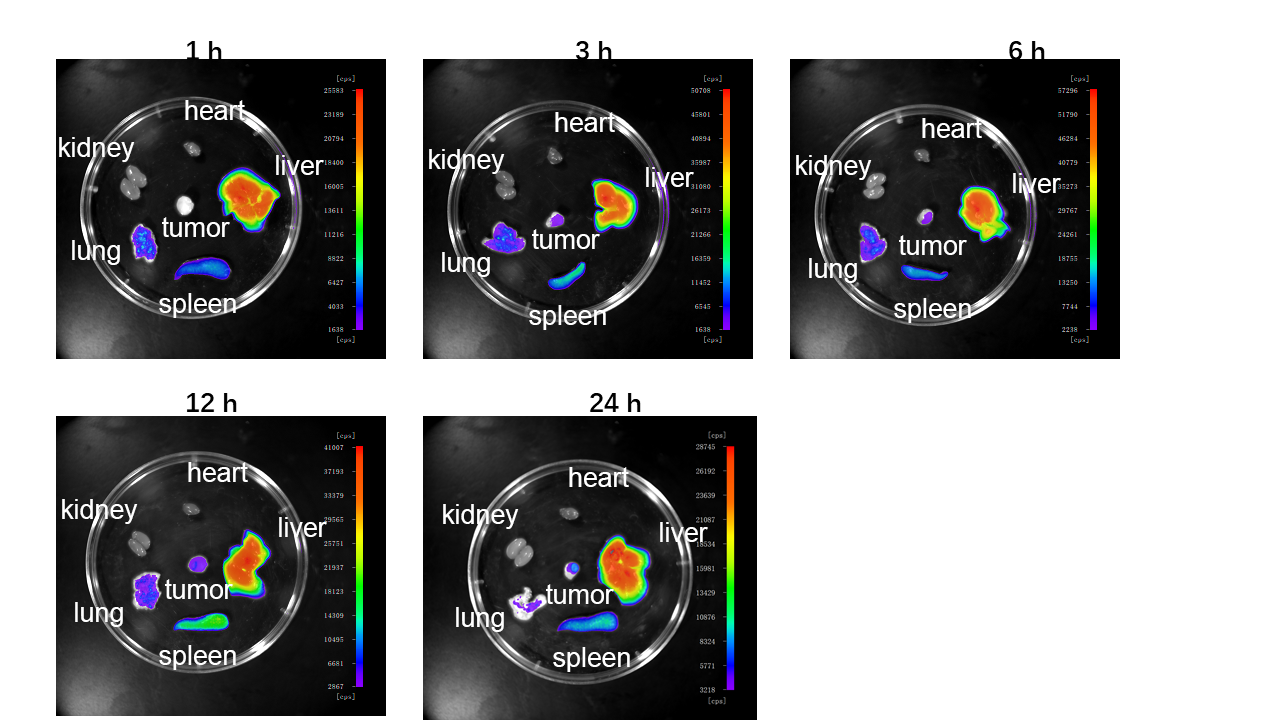


Figure S14 Fluorescence images of the mice major organs and tumors after injection with APP NPs for 1, 3, 6, 12, and 24 h.


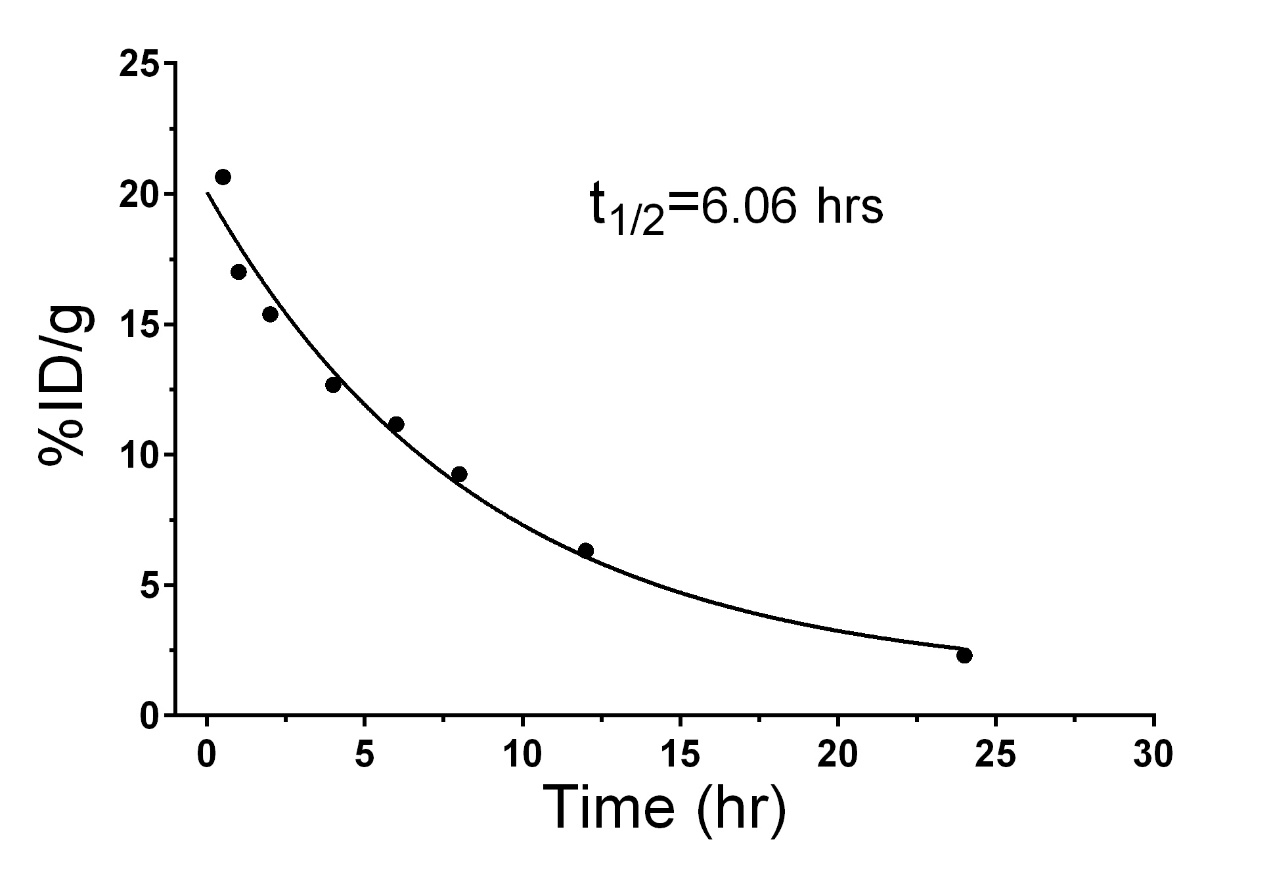


Figure S15 *In vivo* pharmacokinetics of APP NPs in blood after intravenously injecting our APP NPs (10 mg/Kg) into tumor-bearing mice. The content of Zr element in blood at different time points (0.5, 1, 2, 4, 6, 8, 10,24 h) was determined by ICP-MS.


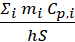


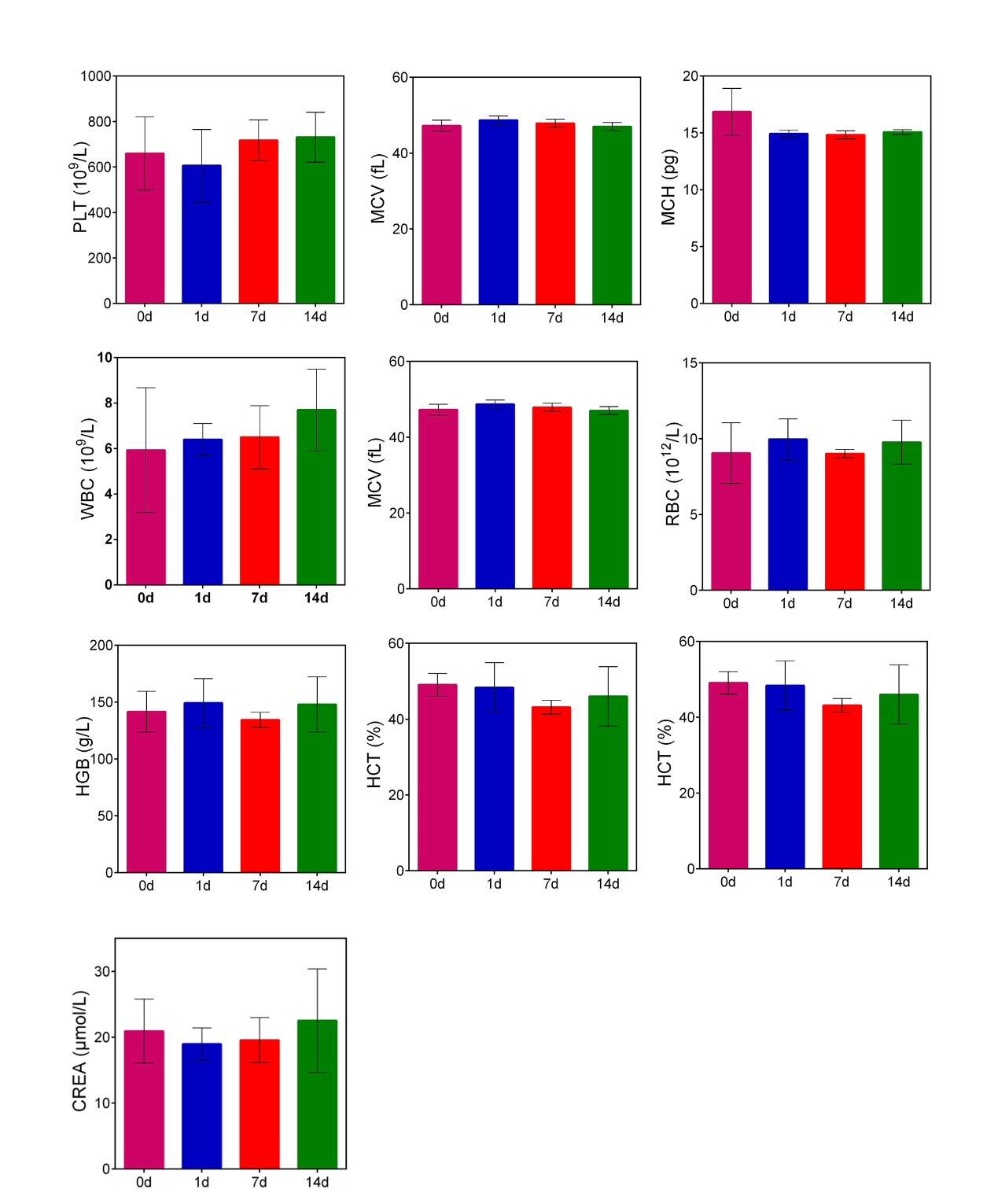


Figure S16 Routine blood parameters and Creatinine (CREA) indexes examination of mice after intravenous injection with APP NPs at different time intervals (ns, no significance).


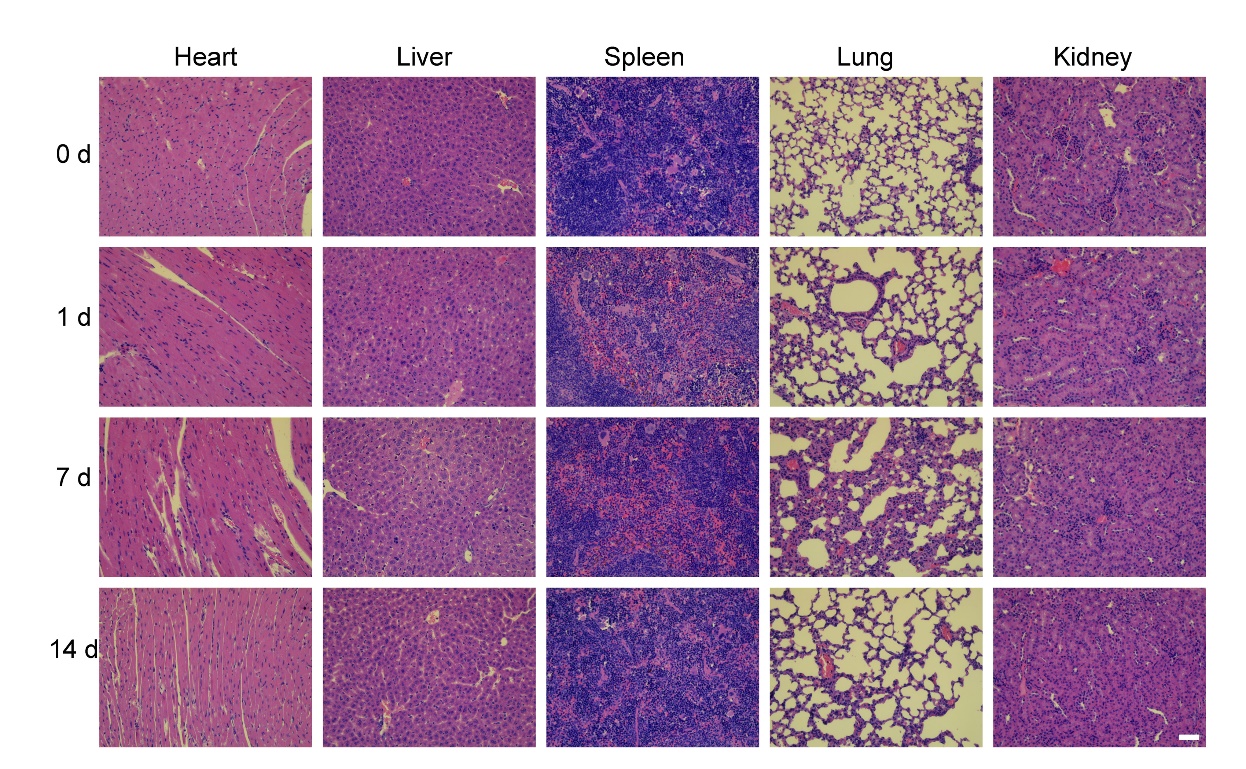


Figure S17 H&E staining images acquired form the major organs (heart, liver, spleen, lung, and kidney) of mice after intravenous injected with APP NPs at different time intervals (Scale bar: 50 μm).


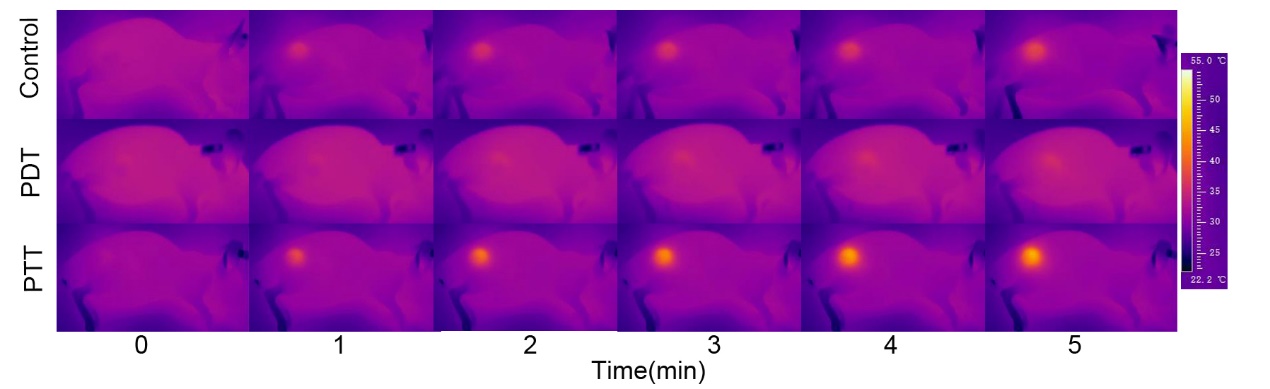


Figure S18 Infrared thermographic images of tumors after treatment with APP NPs under 660 nm laser exposure for PDT and PTT.


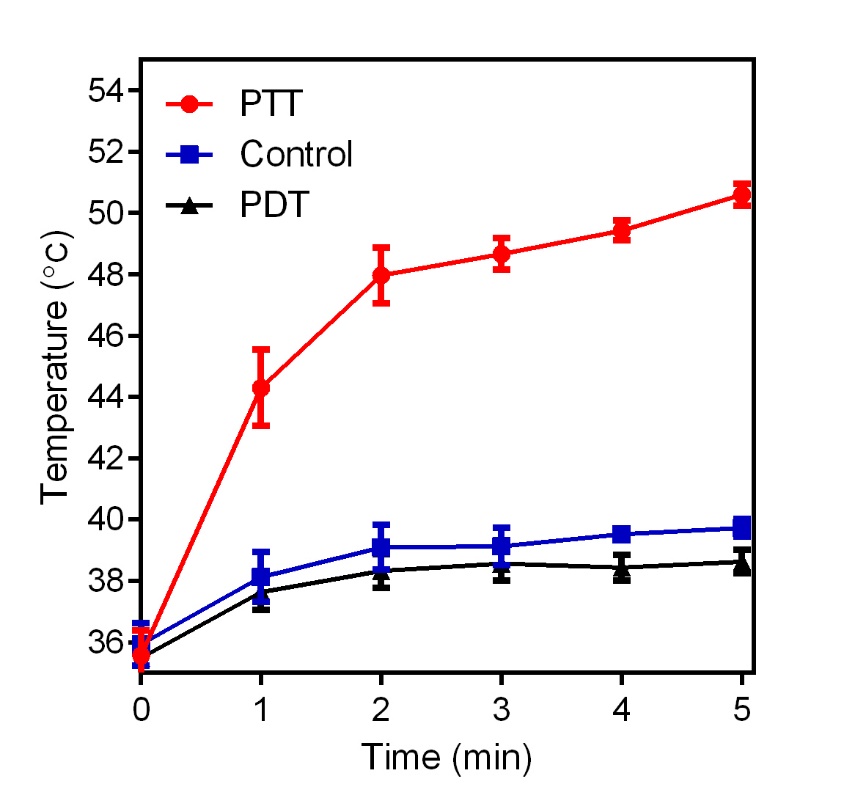


Figure S19 Temperature-elevation curves of tumors after treatment with APP NPs under 660 nm laser exposure for PDT and PTT.


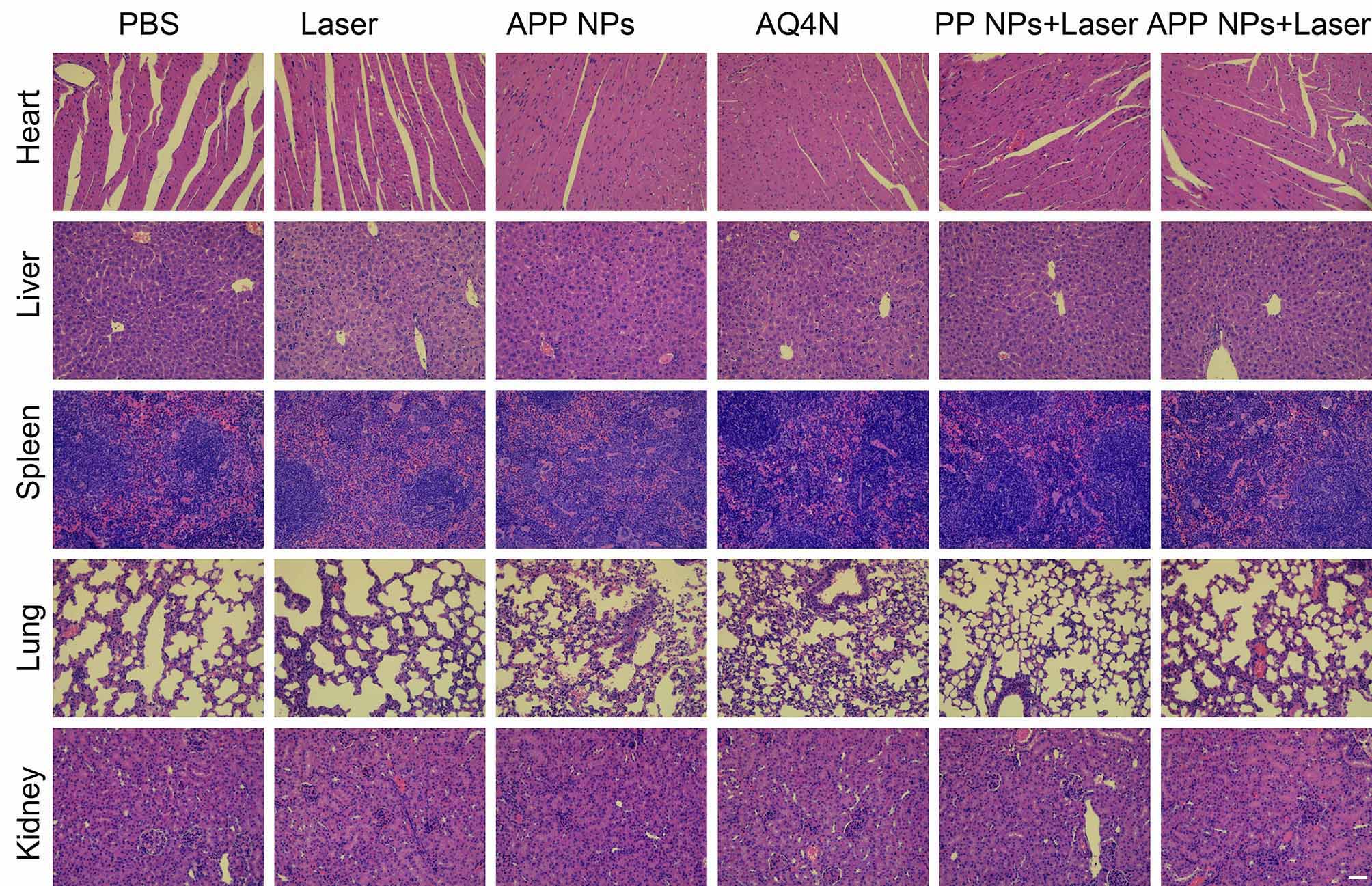


Figure S20 H&E staining images of major organs (heart, liver, spleen, lung, and kidney) acquired for 4T1 tumor-bearing mice after various treatments (Scale bar: 50 μm).


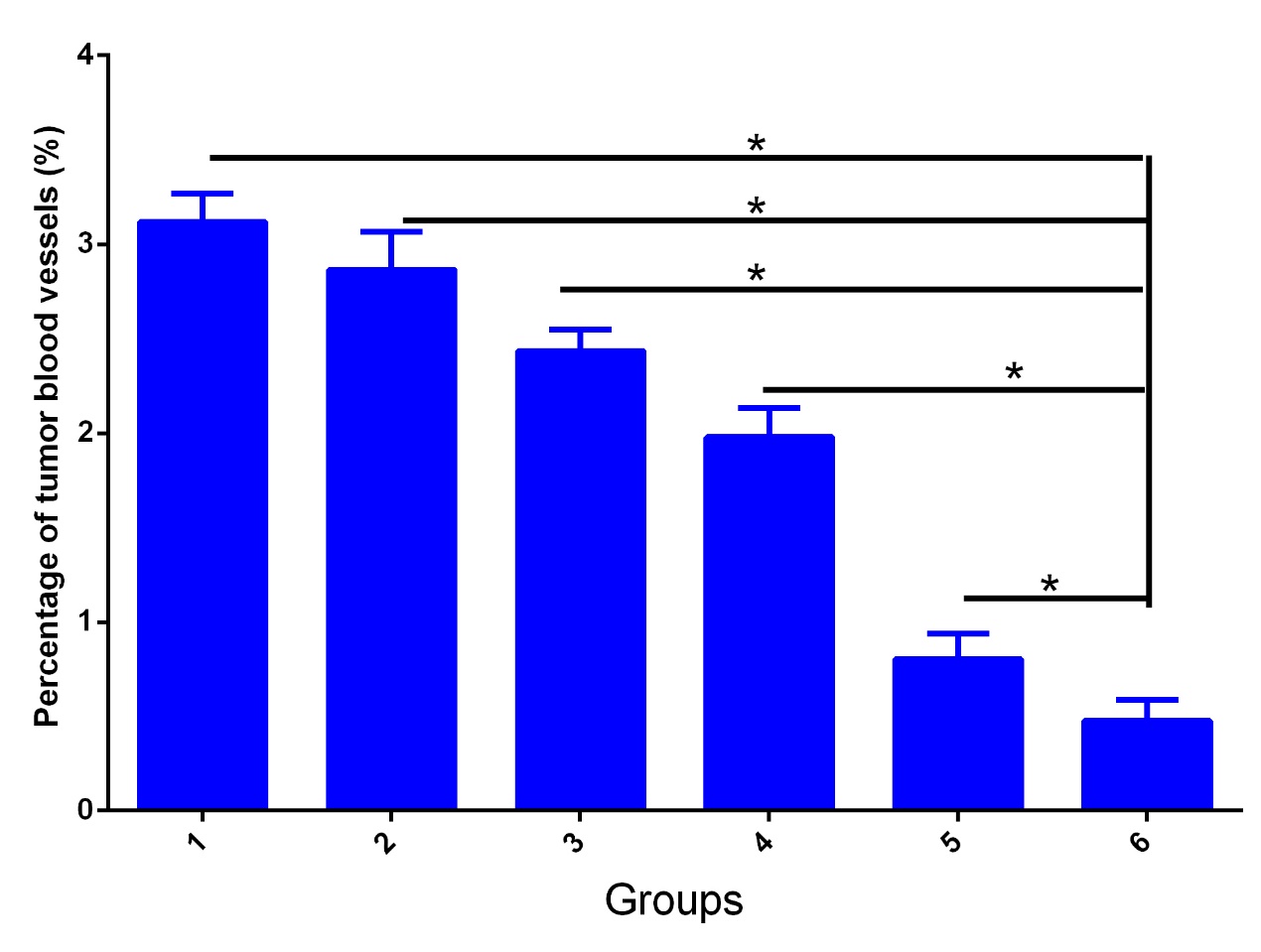


Figure S21 Quantitative analysis of blood vessel area in Figure 8a (CD31) using Imaging J, (n=3, *p < 0.05).
